# Syllabic units in children’s word recognition

**DOI:** 10.3389/fpsyg.2026.1852797

**Published:** 2026-09-10

**Authors:** Devin M. Kearns, Hyelin Jeung, Jeffrey Brown, Yan Wei

**Affiliations:** 1 Department of Teacher Education and Learning Sciences, North Carolina State University, Raleigh, NC, United States; 2 Department of Counseling and School Psychology, San Diego State University, San Diego, CA, United States; 3 Department of Inclusive Education and Behavior Science, Southern Connecticut State University, New Haven, CT, United States

**Keywords:** decoding, polysyllabic words, reading, syllables, word recognition

## Abstract

When elementary-aged children read unknown polysyllabic words, they must parse the orthographic input in order to decode it. Prior studies have not shown clearly whether syllables—as opposed to letters or morphemes—are perceptual units, particularly in children. Children in second, third, and fourth grade (*N* = 123) with and without reading difficulty (RD) were asked to read polysyllabic pseudowords (*N* = 98), and their accuracy, response time (onset latency), and response length (fluency) were recorded. In explanatory item-response analyses, the number of syllables in a word was negatively associated with pronunciation accuracy, latency, and fluency, even when accounting for the number of letters and morphemes. A syllable-by-grade interaction for onset latency showed that the syllable effect was smaller in higher grades, suggesting ongoing development of automaticity with syllabic features. A syllable-by-RD interaction for onset latency and fluency showed a smaller syllable effect for students with RD, suggesting that polysyllabic word processing is challenging for these students at all syllable lengths. The presence of negative syllable effects when accounting for the number of letters and presence of morphemes suggests that syllables are unique sublexical units that require reader attention when pronouncing unfamiliar written words. The mechanisms posited to explain these large negative effects include the need to parse printed words into orthographic syllables, inconsistency in the pronunciation of vowels in polysyllabic words, and the need to assign lexical stress. Overall, the results provide strong evidence that words’ syllabic features require processing that makes words with more syllables harder for elementary-age readers, regardless of the number of letters in the words, and that the degree to which syllabic features impact student performance depends on their age and reading ability.

## Introduction

The issue of interest in this paper is the relationship between the number of syllables in English words and children’s performance in reading—that is, naming—them. This is a practically important issue because late-elementary age children begin to encounter large numbers of polysyllabic words (e.g., Carlisle et al., 2015; Kearns, 2015): Overall, 91% of word types and 27% of tokens readers encounter are polysyllabic (Fitt, 2001; Zeno et al., 1995). It is therefore important to understand whether it is useful to teach children to attend to syllabic units in polysyllabic words. Answering this question is particularly important because many programs to help children with reading difficulty include instruction on and practice with syllabic units (e.g., the long-used *Orton-Gillingham* approach, Gillingham and Stillman, 2014, and other similar programs, e.g., Clark-Edmands, 2012; Wilson, 2005).

This issue also has implications for theories of perception in word reading. Overall, data indicate that readers are sensitive to the statistical properties of the printed words they encounter such that they read words with patterns they encounter more often with greater accuracy and fluency (Seidenberg and McClelland, 1989), but there has been less focus on whether readers–especially children and those with reading difficulty–attend to the syllabic structure of the word when reading it in English. This is particularly important in English–a quasiregular orthography–because polysyllabic English words contain complexities that monosyllabic words do not (e.g., stress assignment; Arciuli et al., 2010; Rastle and Coltheart, 2000). Researchers have not clearly established whether reading accuracy and speed are associated with the number of syllables in the word (Conrad et al., 2007). Most existing data on the importance of sublexical orthographic features address the question whether readers examine unfamiliar words using letter-sounds—grapheme-phoneme correspondences (GPCs)—or body-rime units (Wiley et al., 2023; for data favoring GPCs, see Coltheart and Leahy, 1992; Compton, 2000; Vousden et al., 2011; for body-rime data, see Glushko, 1979; Goswami, 1986, 1988; Treiman et al., 1995; Ziegler and Goswami, 2005). Most of these studies, however, focused on monosyllabic words, presumably on the assumption that their results are relevant for polysyllabic words.

However, the units used to read familiar polysyllabic words may be different. They have features that monosyllabic words do not have (e.g., reduced vowels) and phonemes that are not used in monosyllabic words pronounced in isolation (e.g., /Ɂ/ and /ɾ/). Moreover, some data suggest that readers do not think about the pronunciation of vowels in monosyllabic words and polysyllabic words in the same way (Kearns et al., 2016). Thus, data from studies focused on monosyllabic words paint an incomplete picture of factors that influence word reading skills. One unresolved issue is whether readers use syllables as perceptual units to parse orthographic input when they encounter polysyllabic words. We use the term *perceptual unit* functionally in the sense of syllable-level word structure, particularly in comparison to letters and morphemes. We are not making claims about the internal structure of a putative discrete syllabic chunk.

Several facts suggest that English readers are influenced by syllabic features during reading. First, polysyllabic English words have some apparently reliable orthographic features (e.g., gemination; Venezky, 1999). Moreover, data from other alphabetic languages show clear syllable effects (e.g., for Spanish, Carreiras et al., 2005; for French, Ferrand et al., 1997; Tossonian et al., 2020). In English, it is also true that GPCs are notoriously unreliable given the complex origins of its orthographic system (Balmuth, 1982; Kearns, 2020), so larger orthographic units like syllables may be helpful in the same way as body-rime units if they are more consistent than GPCs (Treiman et al., 1995). To wit, cross-linguistic data indicate that English speakers read words at a larger grain size than readers of consistent languages like Greek (Goswami et al., 1997), and some data show that English speakers even show a tendency to read words at the lexical level (e.g., Rau et al., 2015). Syllables might improve reading performance in the same way as body-rime units. Readers might parse printed polysyllabic words into syllabic units and use the unique information available at the syllable level (i.e., that not available at the letter and grapheme levels) to support accurate and fluent pronunciation. Should this be the case, the number of syllables in a word (the factor considered in the present study) would be associated with better performance perhaps by providing partly redundant orthographic information that over-determines pronunciation. Alternatively, each syllabic unit could introduce additional processing requirements beyond the processing of letter or morpheme units and therefore syllabic units could impede fast and accurate pronunciation.

Therefore, the purpose of this study was to examine whether the number of syllables in polysyllabic pseudowords affects elementary-age readers’ response accuracy, fluency, and onset latency. The goal was to determine whether the number of syllables in pseudowords relates to these outcomes and the direction of these effects, when accounting for effects related to other units.

### Possible importance of syllable-like units in polysyllabic word reading

Many studies conducted with adults have suggested that the number of syllables in words affects reading RTs. Eriksen et al. (1970), Ferrand and Segui (1998), Muncer and Knight (2012), Muncer et al. (2014), Taft and Forster (1976), and Vazeux et al. (2020) have all produced evidence supporting the idea that syllable-level information is used in reading polysyllabic words. Ashby (2006) and Ashby and Rayner (2004) even observed a syllable effect in eye-tracking studies of silent reading, showing that readers had shorter RTs when words were preceded by a syllabically congruent parafoveal preview.

Data from Mewhort and Beal (1977) provide perhaps the clearest evidence that syllable units are important. Their experimental design was unique: They asked adult participants to identify words shown one-by-one in syllabic or nonsyllabic units (e.g., *hos-pi-tal* or *ho-sp-ital*) at different inter-stimulus intervals (ISIs). For longer ISIs (250 ms to 625 ms), participants were less accurate in naming words presented in nonsyllabic units than syllabic units. They also presented the syllables in backward order, i.e., with the last syllable first, and found that these data supported the argument in favor of syllables. These data are particularly compelling because the researchers actually manipulated whether units of similar size were syllables or simply non-syllabic letter combinations. Similar effects were observed in a later, similar study conducted with children by Shefelbine and Calhoun (1991).

What none of these studies addresses, however, is what exactly is meant by a syllable. Because English is a stress-timed language, its phonology requires vowel reduction, unlike syllable-timed languages like French (Ferrand et al., 1996; Ferrand and New, 2003; New et al., 2006). The resulting challenge is that phonological syllables have ambiguous boundaries (e.g., whether *s* in *master* is in the first or second syllable; cf. Clements and Keyser, 1983; Kessler and Treiman, 1997; Treiman and Zukowski, 1990). In addition, orthographic syllable divisions given by dictionaries generally involve placing lax vowels in the middle of syllables (e.g., *i* in *winter, win-ter*) and tense ones at the end (e.g., *i* in *minor, mi-nor*), whether or not this matches the phonology. Thus, what constitutes an orthographic syllabic unit is unclear. However, readers may still perceive some type of *syllable-like unit*. Researchers have put forth several different ideas about the nature of the salient unit.

Vocalic center groups (VCGs; Hansen and Rodgers, 1965) are orthographic units anchored by the letters associated with vowel phonemes (*A, E, I, O, U*, or *Y* when not in a consonant position). Spoehr and Smith (1973) and Chetail and Content (2014) have produced data suggesting that readers parse words according to these units. Different instantiations by Taft (1979, 1992) have also shown that a syllable-anchored unit is critical for perception and emphasized the importance of including all orthotactically legal consonants in a word’s first perceptual unit, what he called the basic orthographic syllabic structure (BOSS; e.g., *lant* in *lantern*). He considered a slight alternative, the body of the BOSS (BOB; e.g., *ant* in *lantern*; Taft, 1992) and appeared to show that this was a more important perceptual unit than the BOSS. The BOSS and BOB differ in whether the orthographic onset is an important unit, but the results remain consistent with the idea that either vowel-anchored syllable-like structure is a perceptual unit that provides access to the word’s lexical entry. Lima and Pollatsek (1983) provided further data that a single vowel should anchor the unit with data showing that adding a vowel following the BOSS (e.g., *burde* versus *burd* for *burden*) resulted in longer RTs than BOSS units or VCGs. Whatever their differences, all of these instantiations of syllable-like orthographic units result in the conclusion that readers perceive a structure containing an orthographic vowel when they parse polysyllabic words.

In the present study, we examined whether elementary-aged children exhibit the same attention to a syllable-like vowel-anchored orthographic unit shown in adults reading completely unfamiliar words (i.e., pseudowords). Certainly, the findings described above suggest that children would exhibit attention to syllables, but their processing is quite likely to differ from adults’.

It is not clear whether syllable-related findings from adults would follow the same pattern for elementary-age children with and without reading difficulty and in English. One possibility is that children process written input at the letter level without attention to the internal orthographic consistencies. In English orthography, (almost) every syllable requires inclusion of at least one of six vowel letters; in other words, the orthography encodes syllabic features. However, early readers may not process words using this information: Words might be perceived letter-by-letter such that the vowel-anchored syllabic structure has little relevance in linking letters to phonological units. It fits a computational model in that the incremental nature of reading acquisition means that the reading network improves in early acquisition using the frequent and consistent knowledge available at the letter level, especially for consonant letters; later tuning of the reading network will involve the more subtle, contextual, and multi-letter patterns related to syllables (e.g., Harm and Seidenberg, 2004) that are relevant for fewer words but allow access to a larger set of words in the lexicon (Perfetti, 1992; Share, 1995). Put differently, children may not have developed the sensitivity to syllable-like structures that is characteristic of adults.

As a result, our focus is whether words with more syllables are pronounced less accurately and more slowly than words with fewer syllables and whether an observed syllable effect is robust to simultaneous analysis of letter effects in a series of regression models. If syllables add processing complexity, it would require less effort to read words with fewer syllables than those with more, and this effect would be robust to the presence of letters. The alternatives are that syllabic effects are either null when accounting for the number of letters or the presence of morphemes or support better performance due to the additional orthographic information they provide readers.

### The importance of letters in polysyllabic word reading

As described above, a syllable effect would only be meaningful if it were present when controlling the number of the letters in a word. Data certainly support the idea that letters are important perceptual units in word recognition, especially during reading acquisition (e.g., Compton, 2000; Hudson and Bergman, 1985; Marmurek, 1997; Martens and de Jong, 2006; Mason, 1978; Weekes, 1997), but studies where researchers test the relative importance of letters and syllables are less common. New et al. (2006) used the English Lexicon Project adult naming accuracy and RT data and showed that longer words had shorter RTs if the number of letters was less than 6, that letter length had no effect if the number of letters was between 5 and 8, and that each additional letter increased RTs for words with 9 or more letters. Yap and Balota (2009) replicated this effect with a different subset of the English Lexicon Project data. The longer-word data are especially relevant for the present study because the majority of words (81 out of 98) had at least 7 letters. Moreover, the letter effect for short words probably reflects the competition between short words with necessarily similar orthographic patterns (orthographic *N* and orthographic Levenshtein distance effects; e.g., Andrews, 1997), and words with fewer than 6 letters are infrequently polysyllabic.

In the English Lexicon Project studies by New et al. (2006) and Yap and Balota (2009), they also observed that adults had longer RTs for words with more syllables even when controlling for the number of letters. In the few experimental studies that attempted to separate letter and syllable effects, the adult participants showed sensitivity to the number of letters but not the number of syllables (Frederiksen and Kroll, 1976; Forster and Chambers, 1973; Richardson, 1976).

There is only one study in which researchers have examined whether children’s ability to read polysyllabic words depends on the number of syllables or letters it contains. Hautala et al. (2013) examined these effects in good and poor readers in Finnish: Good readers appeared to rely on syllabic units to read words while poor readers depended on letters, graphemes, or consonants, with the idea that sensitivity to syllabic units is acquired after acquisition of letter-sound consistencies. Overall, however, extant data are equivocal whether syllables, letters, or both are key units, so our test of the syllable effect in the presence of the letter effects is an important one. Moreover, many of these results were observed in languages with more transparent orthographies than English (an outlier orthography; Share, 2008), and various syllable-related features of the language (e.g., the single-letter vowels in polysyllabic words with highly variable pronunciations; e.g., Kearns, 2020; Rastle and Coltheart, 2000) are almost certain to affect performance in English–raising the possibility that syllable effects in English are more complex than in other orthographies.

### The importance of morphemes in polysyllabic word reading

Morphemes might be another salient unit that could compete with the syllable. Data consistently show that morphemes affect children’s word reading skill (e.g., Apel et al., 2012; Carlisle, 2000; Deacon, 2012; Goodwin et al., 2013; Kearns, 2015; Levesque et al., 2019; McCutchen et al., 2008; McCutchen et al., 2009; Singson et al., 2000). In terms of the hypothesis concerning syllables, the possible alternative is that effects perceived as syllabic might simply reflect attention to morphological information. Syllables are often consistent with morphemes (e.g., *unhelpful*) but not always (e.g., *bitterly; derivation*). It is possible that readers simply perceive morphemes (e.g., −*ation*) and use them. Thus, the number of morphemes would matter but the number of syllables would not. In the few studies that have examined the relation between morphology and polysyllabic polymorphemic word reading, these possibilities have not been compared. Goodwin et al. (2013) tested neither letter nor syllable effects on adolescents’ accuracy reading polymorphemic words. Kearns (2015) tested whether the number of letters affected polymorphemic word reading accuracy (it did not), but syllable effects were not considered. De Simone et al. (2023) studied syllabic and morphological processing in German adult readers using an eye-tracking paradigm and observed that visual disruption of syllable boundaries resulted in longer fixations than disruption of morpheme boundaries. These studies leave open the possibility that syllables are the more important units, despite their results favoring the idea that morphemes are key to word reading.

Some experimental studies have addressed the importance of morphological effects in polysyllabic words, directly contrasting their effects. Carlisle and Stone (2005) showed that elementary-age children had faster responses to same-length words containing morphemes than those without them (e.g., *shady* versus *lady*). Prinzmetal et al. (1986) used an illusory conjunction paradigm to test whether adult participants erred in reporting the color of letters based on morphological or syllabic information (e.g., *x* as the color of *o* or *l* in *loxly* versus *z* as the color of *g* or *i* in vizgo*). They concluded that both effects were present: There was a strong morphological effect, but there was a similar effect based on orthographic syllables. Thus, the data from Carlisle and Stone (2005) seem to contrast with those from Prinzmetal et al. (1986), and no other studies have directly examined both effects. In summary, equivocal prior data on the role of syllables and morphemes in understanding word processing motivated the present study to examine the effects of both simultaneously. We examine the possibility that readers focus on morphemes rather than syllables when reading English polysyllabic words.

### Attention to multiple units simultaneously

It may be that none of these three units dominates the process of reading an unknown word; knowledge acquired about syllables, letters, and morphemes may all have relevance. In a parallel distributed processing (connectionist) account of word reading, readers draw on multiple sources of information in words simultaneously (Plaut et al., 1996). So far, we have emphasized that either syllables or morphemes are salient orthographic units without considering the possibility that both may be important. In a parallel distributed processing model, readers do not represent syllables or morphemes as separate sublexical components but build representations that depend on the statistical regularities in the words the reader encounters. The links that develop between letters and sounds can occur at multiple levels and simultaneously contribute to fast accurate word reading. The Connectionist Dual Process (CDP++) model tested by Perry et al. (2010) resulted in longer latencies for bisyllabic words than monosyllabic words, suggesting that the complexities introduced by the additional syllable slowed the network’s response times (in cycles needed to reach criterion). This result provides some evidence that the syllable effect exists and is negative, with each additional syllable reducing accuracy and increasing latency, regardless of letter-related processing demands. This result was further supported by their observation that letter-sound consistency reduced response times. The fact that these consistencies were calculated at the letter level also suggests that letter-level information contributed separately to performance. Therefore, in the current study, there is the possibility of syllable effects when we account for the number of letters and the presence of morphological units—and that we will observe letter and morpheme effects as well.

### Present study and research questions

In summary, this study was designed to address the question whether elementary-age children use syllables as perceptual units in reading unknown words, whether they instead rely on letters or morphemes to read these words, or whether children show sensitivity to all three units. Specifically, the present study investigated elementary children’s response accuracy, fluency, and onset latency for reading polysyllabic pseudowords varying in the number of syllables, number of letters, or number of morphemes. These are the questions:

#### Research question 1: syllable effects alone

What is the effect of the number of syllables on children’s (a) accuracy, (b) fluency, and (c) onset latency for reading polysyllabic pseudowords? It was hypothesized that children would read words with more syllables less accurately, less fluently, and with longer onset latency compared with words with fewer syllables due to the increased complexity introduced by each additional syllable.

#### Research question 2: syllable effects with letter effects

Is the number of syllables a significant predictor of these outcomes when accounting for the number of letters? We expected that words with more letters would be read less accurately, with longer response times, and for a longer period of time compared with those with fewer letters. We expected that the additional complexity involved in processing words with additional letters would have a robust effect as in prior studies.

In terms of the hypothesis, the direction of the syllable effect could be positive or negative. Framed as a facilitative effect, the additional syllable would provide additional information to support word recognition in that written syllables are orthotactically coherent units the reader might encounter multiple times. Because multi-letter units generally provide more consistent information than individual letters, syllabic units would reinforce the consistencies in letter-level pronunciations and serve to reduce overall variability (e.g., Perfetti, 1992). The alternative prediction is that the syllabic unit introduces demands like stress assignment not required when reading monosyllabic words. Thus, each additional syllable simply compounds the challenge of letter-level processing. We were especially interested in the fluency finding because the length of a response is necessarily associated with the length of the word, but the letter-length covariate would produce a net null effect for the syllable if there were no unique syllable factor.

#### Research question 3: morphological effects

Is the number of syllables a significant predictor of these outcomes when accounting for a wider range of competing effects? The RQ3 model included morphological covariates, operationally defined in terms of the presence of a known affix or real word at the beginning or end of the pseudoword. We expected that performance on words containing morphological information would be better than for words without these features because morphemes are especially high-frequency orthographic units with consistent phonological associations. However, prior data did not provide adequate guidance to form a clear hypothesis concerning morpheme effects when also considering syllable effects.

#### Research question 4: grade and RD status effects

Do syllable effects vary depending on student grade or RD status? We hypothesized that students in Grade 3 would have lower onset latency, more fluent responses, and greater accuracy than students in Grade 2. We also expected that these effects would interact with the syllable effect such that Grade 2 students would have worse performance than expected compared to their Grade 3 counterparts because Grade 2 students would have had less exposure to longer words and the additional complexity of three-syllable words would further impede their ability to process these words. We expected that the difference in performance of two- and three-syllable words between students with typical achievement and their counterparts with RD would follow the same pattern as performance between Grade 2 and Grade 3 students.

## Method

### Participants

Students in Grades 2, 3, and 4 participated in the study (*N* = 123; see Table 1). All Grade 2 students demonstrated typical achievement (TA), whereas the Grade 3 and Grade 4 groups included both students with TA and reading difficulties (RD; see Table 2). Reading difficulty status was calculated based on readers’ composite standard scores for the Sight Word Efficiency (SWE) and Phonological Decoding Efficiency (PDE) subtests of the *Test of Word Reading Efficiency, 2nd Edition* (TOWRE2; Torgesen et al., 2012). Children whose TOWRE2 composite standard scores were below the 25th percentile for the student’s grade level were considered to have reading difficulty. Those whose standard scores were above the 35th percentile were considered to show a TA profile. The second-grade group was selected to approximate a reading age-match with the children with reading difficulty, meaning that they had to have TOWRE2 scores above the 35^th^ percentile using the second-grade norms but below the 25^th^ percentile using the third-grade norms. Participants were from five schools in three school districts in the northeastern United States. The study was approved by the Institutional Review Board of the principal investigator’s employer. Prior to enrollment in the study, parents received a permission form written in non-technical language and signed it if they wished. Prior to beginning any assessment with a student, the test examiner provided the student with a spoken explanation of the activities involved in participation in non-technical language and provided an opportunity to ask questions. The child would provide verbal assent if they wished to participate. For analysis, grade was coded as a continuous variable with second grade as the intercept value; RD status was coded dichotomously with TA as the intercept value. Descriptive statistics for participants are presented in Table 1.

**Table 1:** Child demographic characteristics (*N* = 123).

Variable	Category	*n*	%
Grade	Second	23	18.7
	Third	53	43.1
	Fourth	47	38.2
RD status	RD	51	41.5
	TA	72	58.5
Gender^a^	Male	53	45.7
	Female	63	54.3
Race/Ethnicity^a^	African-American/Black	9	7.8
	Asian-American	10	8.6
	White	86	74.1
	Hispanic/Latino	4	3.5
	Multiethnic	4	3.5
	Other Category	3	2.6
Special education status^a^	Does Not Have IEP	93	80.2
	Has IEP	20	17.2
	In Referral Process	3	2.6
		

^a^Data for these demographic variables were only available for 116 children. IEP = Individualized Education Program.

**Table 2:** Distribution of reading difficulty across grades.

Reading difficulty	Grade			Total
	2	3	4	
No RD	23	22	27	72
RD	0	31	20	51
Total	23	53	47	123

### Polysyllabic pseudoword reading measure

Words (*N* = 98) were chosen because they included the syllables from target words for another experiment (Kearns and Al Ghanem, 2019). All target words included derivational morphemes. In the construction of the pseudowords, one of the syllables was changed to construct the pseudoword following the procedure used by the Wuggy pseudoword generator (Keuleers and Brysbaert, 2010). Three outcomes were measured, accuracy, fluency, and onset latency. Responses were coded using recordings of children’s pronunciations. The research team created a comprehensive coding manual that described what constituted an accurate, fast, and fluent response. Three raters independently coded 10% of all tests and their interrater reliability was calculated by checking each pair of raters and averaging the three ratings (*κ* = 0.91).

Missingness was also examined; there were 12,054 possible responses (child *N* = 123; word *N* = 98), but 4% of responses were inaudible, resulting in 11,523 responses available for analysis. The missing data were assumed to be missing conditionally at random. In other words, whether responses were inaudible on specific items would not relate to the items themselves, but specific children might produce more inaudible responses than others due to some child-specific factor. However, we judged that this did not affect the prediction of word features, particularly because the rate of missingness was so low.

In the following sections, descriptions of the dependent and independent variables are given. Descriptive statistics for student performance are given in Table 3, and descriptive statistics and bivariate correlations for all word variables appear in Tables 4, 5.

**Table 3:** Descriptive statistics for child responses (*N* = 123) for pseudowords (*N* = 98).

Variable	Intercorrelations			*M*	*SD*	Min	Max
	1	2	3				
1. Accuracy (response coded as correct)	—			0.41	0.49	0.00	1.00
2. Fluency (response duration is less than 2 s)	0.12	—		0.73	0.44	0.00	1.00
3. Onset latency (started within 2 s)	0.07	0.12	—	0.76	0.43	0.00	1.00

All correlations are significant at *p* < 0.001.

**Table 4:** Descriptive statistics for word variables (*N* = 98).

Variable	Category	*n*	%
Number of letters	5	3	3.1
	6	14	14.3
	7	46	46.9
	8	30	30.6
	9	5	5.1
Number of syllables	2	61	62.2
	3	37	37.8
Starts with real prefix	No	86	87.8
	Yes	12	12.2
Ends with real suffix	No	47	48
	Yes	51	52
Has real word beginning	No	70	71.4
	Yes	28	28.6
Has real word ending	No	60	61.2
	Yes	38	38.8

**Table 5:** Word-level bivariate correlations among word variables (*N* = 98).

Variable	1	2	3	4	5	6	7
1. Number of syllables	–						
2. Number of letters	0.45***	–					
3. Start with real prefix	−0.03	−0.16	–				
4. End with real suffix	0.28**	0.30**	−0.08	–			
5. Has real word beginning	−0.17	−0.02	−0.17	0.02	–		
6. Has real word ending	−0.10	0.13	−0.04	−0.07	−0.04	–	
7. Number of vowels	0.35***	0.49***	0.00	0.17	0.01	0.08	–
8. Bigram frequency	0.36***	0.52***	0.10	0.29**	0.08	0.06	0.08

*p < 0.05, **p < 0.01, ***p < 0.001. Bigram Frequency was log-transformed and mean-centered prior to analysis.

#### Pseudoword response accuracy (accuracy)

Accuracy was judged based on whether the response was plausible. The research team determined which pronunciations were plausible based on the characteristics of the given word and the possible responses for similar real words. In addition, the research assistants checked whether other adults produced responses that met the plausibility criteria. Accurate—that is, plausible—responses were coded 1 and inaccurate (implausible) responses coded 0. Internal consistency was high, *α* = 0.94.

#### Pseudoword response fluency (fluency)

Fluency was determined by the length of the attempted response, that is, whether it was longer or shorter than 2 s. We coded responses that lasted less than 2 s as fluent (coded 1) and those that lasted more as dysfluent (coded 0). Internal consistency was high, α = 0.97.

#### Pseudoword response onset latency (latency)

Onset latency—that is, response time—was determined by the onset of the response time, whether it was within 2 s or not. Responses with onset latency less than 2 s were considered to have short onset latency. This was coded with a 1 because shorter onset latencies indicate a more capable response and allowed us to keep all three measures on the same scale. Internal consistency was high, α = 0.97.

### Word characteristic measures

#### Number of letters (letters)

The number of letters in the words ranged from 5 to 9. For analysis, letter was a continuous variable scaled with the response for 7 letters as the intercept value.

#### Number of syllables (syllables)

The words contained either 2 or 3 syllables. Syllables were identified only by the presence of a vowel sound. The number of syllables was coded dichotomously with bisyllabic word status as the intercept value.

#### Morphological variables (real word beginning, real word ending, real prefix, and real suffix)

We used multiple morphological variables, mainly concerning the presence of real words and affixes. Two variables concerned the presence of real words in the words presented. One was whether the word began with an identifiable real word (Has Real Word Beginning). For example, the word *sweatle* began with the word *sweat*, so this was coded as beginning with a real word. We determined whether it would be considered a real word by children. The other variable was whether the word ended with a real word (Has Real Word Ending).

Two other variables involved affixes. One was whether the word began with a known prefix (Starts with Real Prefix). The other was whether the word ended with a known suffix (Ends with Real Suffix). Each of these was coded dichotomously, with 0 meaning the word did not contain this unit and 1 meaning it did. All of these variables were added as covariates in the morpheme model; we did not create a composite score.

#### Supplemental word variables (number of vowels and bigram frequency)

For our supplemental analyses, we used two additional variables. The number of vowels was defined as the total count of all individual vowel letters (*A, E, I, O, U*, or *Y* when not in a consonant position) present in the word. Bigram frequency was operationalized as the log summed token frequency of bigrams in the words in the Zeno et al. (1995) database for words in texts for students in Grade 1 to Grade 5.

### Procedure

As described above, participants were screened using the TOWRE2 SWE and PDE standard score composite. Children with TOWRE2 scores above the 35th percentile for their given grade were included, and third and fourth grade poor readers with scores below the 25th percentile were also included. The items were randomly ordered and separated into three lists with other unrelated tasks interspersed. The lists were separated to reduce performance fatigue, an especially important factor given that reading stamina is a challenge for younger children and those with reading difficulty. The lists were administered in the same order for all children. We considered randomly ordering the lists but determined that the logistic challenge of creating and distributing randomized assessments was unnecessary. Our rationale was two-fold: First, we judged that the presentation order was unlikely to have an effect because the number of word-characteristic dimensions (letters, syllables, and the four morphological factors) would make the item performance effectively independent. Second, we determined that any performance effect due to presentation order would not correlate with the syllable, letter, or morpheme factors because the words themselves were randomly ordered. In other words, even if child performance was better for later items, the order-based improvement would be distributed across syllable, letter, and morpheme factors unless order differentially affected performance by word characteristic. We judged this unlikely and therefore decided not to randomize the order.

### Data analysis

Data analysis was conducted with explanatory item response models (De Boeck and Wilson, 2004), specifically, an item response model with a random item and a random person parameter (Janssen et al., 2004; Van den Noortgate et al., 2003). The explanatory item response model was constructed to explain the variability in person abilities and item difficulties with item-specific fixed effects for person characteristics (specifically, for grade and RD status) and item characteristics (word features, specifically for syllable, letter, and morpheme). These models are especially useful for examining data where the features of the words and the characteristics of the participants are both important for answering the research questions. In this case, the interactions between child characteristics (e.g., grade) and word features (e.g., number of syllables) motivated this analytic approach. The random effects are necessary because word performance is clustered within each individual child (that is, some children read better than others overall which then affects performance for every word). If each word-level response is treated as an independent observation (as it would be in a typical logistic regression), the model would be misspecified. These models are also valuable because they are robust to unbalanced data in ways that mitigate differences in sample sizes by grade, RD status, and other such factors.

Rasch models, which maintain the assumption that items discriminate similarly across persons (De Boeck and Wilson, 2004), are used here. Analyses used generalized linear mixed-effects modeling for dichotomous outcomes as implemented in the glmer function (version 1.1–38) from the lme4 package in R (Version 4.4.3; Bates et al., 2015; R Core Team, 2025) with BOBYQA optimization (Powell, 2009) and Laplace approximation, an approach validated by Cho et al. (2012) and appropriate for the current data structure (random intercepts only). Our base model included covariates for grade (as a continuous variable) and RD status. Nested χ^2^ tests were used to compare model fit between simpler and more complex models (i.e., in sequence with added syllable, letter, and morphological predictors).

## Results

Table 6 shows the magnitude of the syllable coefficients for successive models for all three outcomes and the changes in deviance and the distribution of random effects that indicate the improvement in model fit. Our first question concerned the effect of the number of syllables on our accuracy, fluency, and onset latency outcomes. There were significant effects for all three outcomes. The coefficients represent the difference in difficulty due to a word having three syllables rather than two for a Grade 2 student without RD. For accuracy, three syllable words reduced the probability of a correct response for that child from 0.31 to 0.18. Three syllable words reduced the probability of a fluent response from 0.68 to 0.44 and the probability of a short onset latency from 0.39 to 0.29.

**Table 6:** Syllable effects and changes in random variability across models.

Model	Syllable effect	Odds ratio	Random effects^a^		Deviance^b^
			Child	Word	
Accuracy					
Base^c^			0.606	0.837	13,206
Syllable only	−0.741***	0.48***	0.606	0.707	13190***
Syllable and letter	−0.523**	0.59**	0.606	0.665	13185*
Syllable, letter, and morpheme	−0.493*	0.61*	0.606	0.640	13,181
Fluency					
Base			1.478	0.573	10,549
Syllable only	−0.986***	0.37***	1.475	0.339	10504***
Syllable and letter	−0.634***	0.53***	1.475	0.228	10472***
Syllable, letter, and morpheme	−0.658***	0.52***	1.475	0.224	10,471
Onset latency					
Base			2.764	0.198	8,652
Syllable only	−0.433***	0.65***	2.764	0.153	8635***
Syllable and letter	−0.308**	0.73**	2.765	0.141	8630*
Syllable, letter, and morpheme	−0.292**	0.75**	2.766	0.137	8,628

The base model contains Grade and reading difficulty status fixed effects. ^a^Random effects are given in SD units. ^b^*p*-values represent significance of χ^2^ test of difference in deviance vs. prior model. ^c^Base model includes grade and reading difficulty covariates. *p < 0.05. **p < 0.01. ***p < 0.001.

The second question concerned the effect of the number of syllables on the outcomes when accounting for the number of letters. The addition of the number-of-letters covariate to the model significantly improved model fit for all three models (see difference in deviance reported in Table 6), suggesting that student performance was affected by the number of letters in addition to the number of syllables. For accuracy, the syllable effect was significant and reduced the probability of a correct response from 0.37 for a bisyllabic word to 0.26 for a trisyllabic word, for the intercept case of a Grade 2 student without RD for a word with seven letters (the median). For fluency, the syllable effect was also significant and reduced the probability of a fluent response from 0.35 for a bisyllabic word to 0.22 for a trisyllabic word. For onset latency, the syllable effect was significant and reduced the probability of a short onset latency from 0.42 to 0.35.

The third question concerned the effect of the number of syllables when we accounted for the effects of letters and morphemes. When the four morpheme-related variables were entered as a block, the difference in deviance between this model and the RQ2 model with only syllable and letter covariates was not significant for any of the three outcomes (see the deviance column in Table 6). As a result, we judged that the model containing syllable and letter covariates was the most parsimonious for these data and did not further explore the role of morphological features in the context of understanding the role of syllables in reading the words.

**Figure 1:**
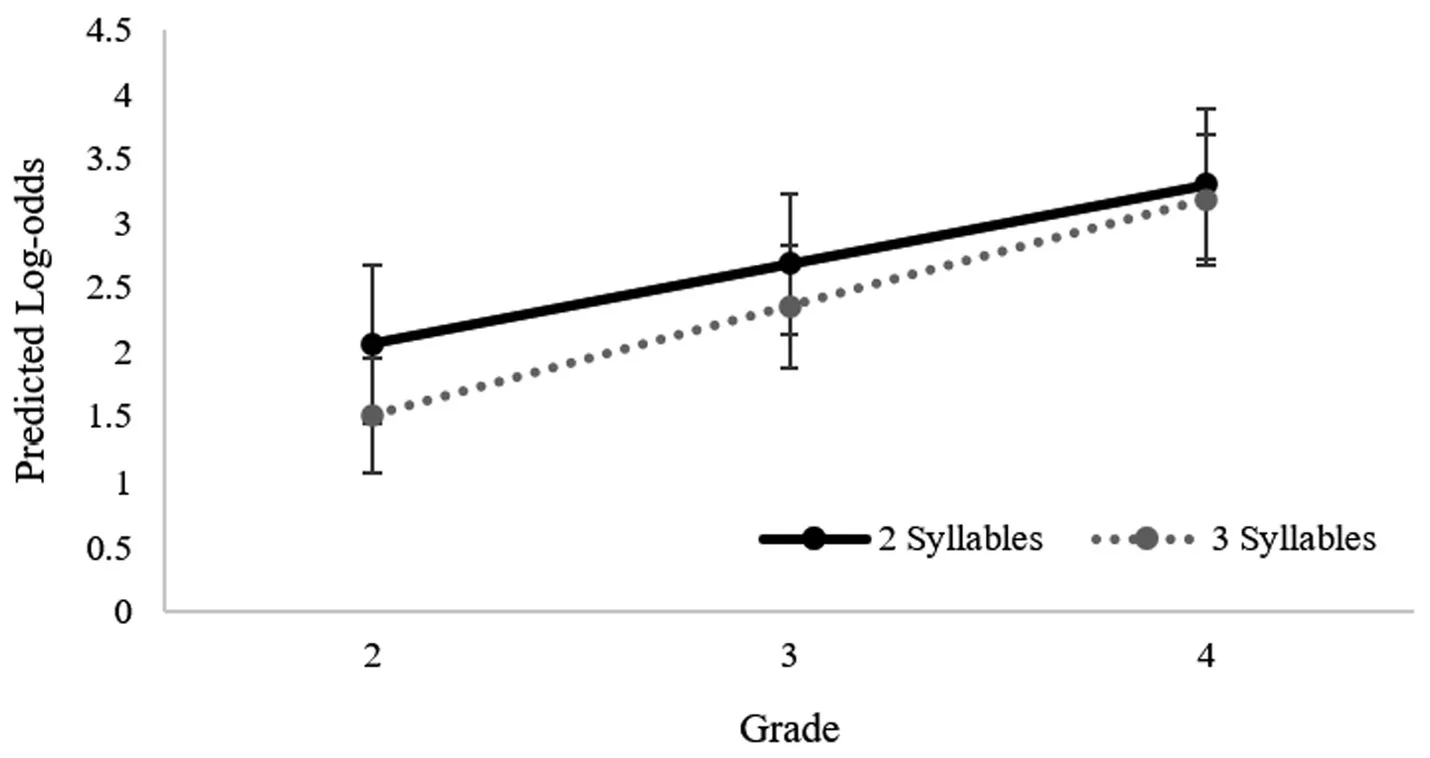
Interaction between number of syllables and grades for onset latency.

**Figure 2:**
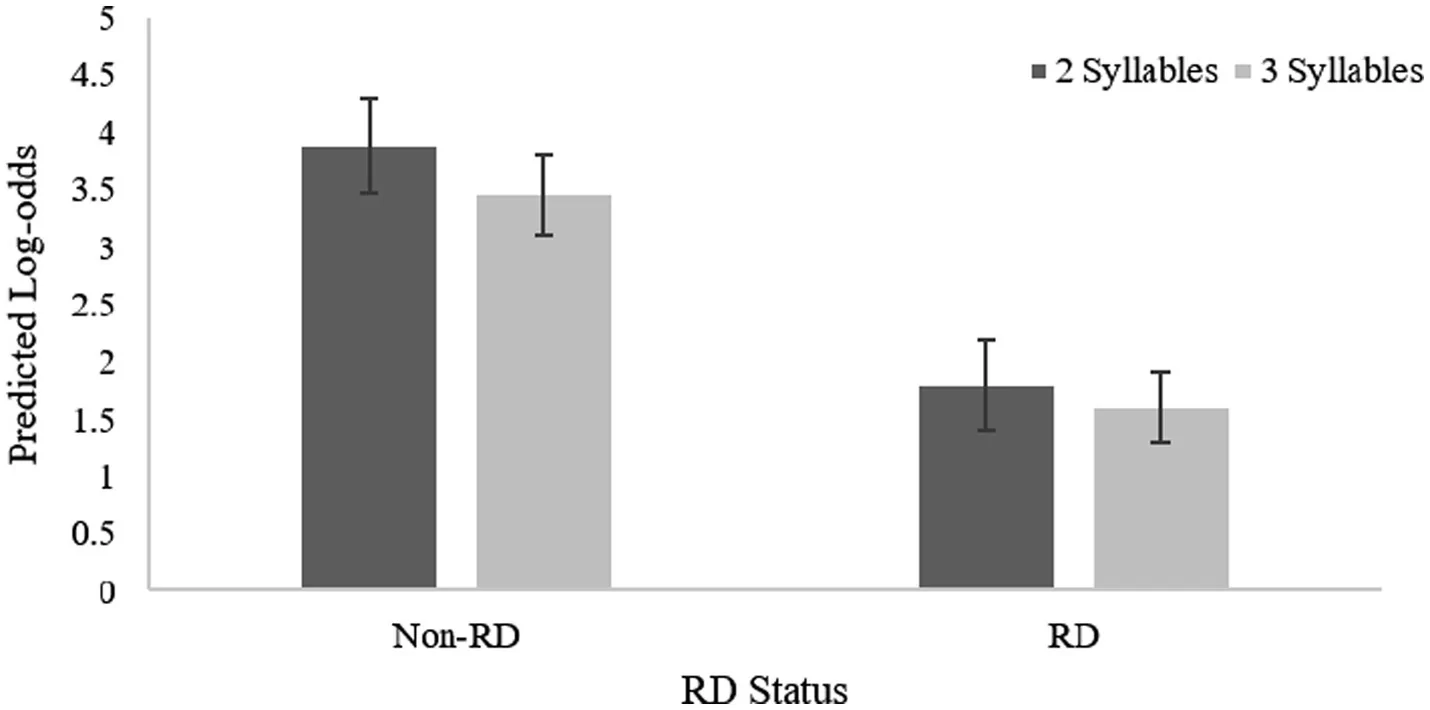
Interaction between number of syllables and RD Status for onset latency.

**Figure 3:**
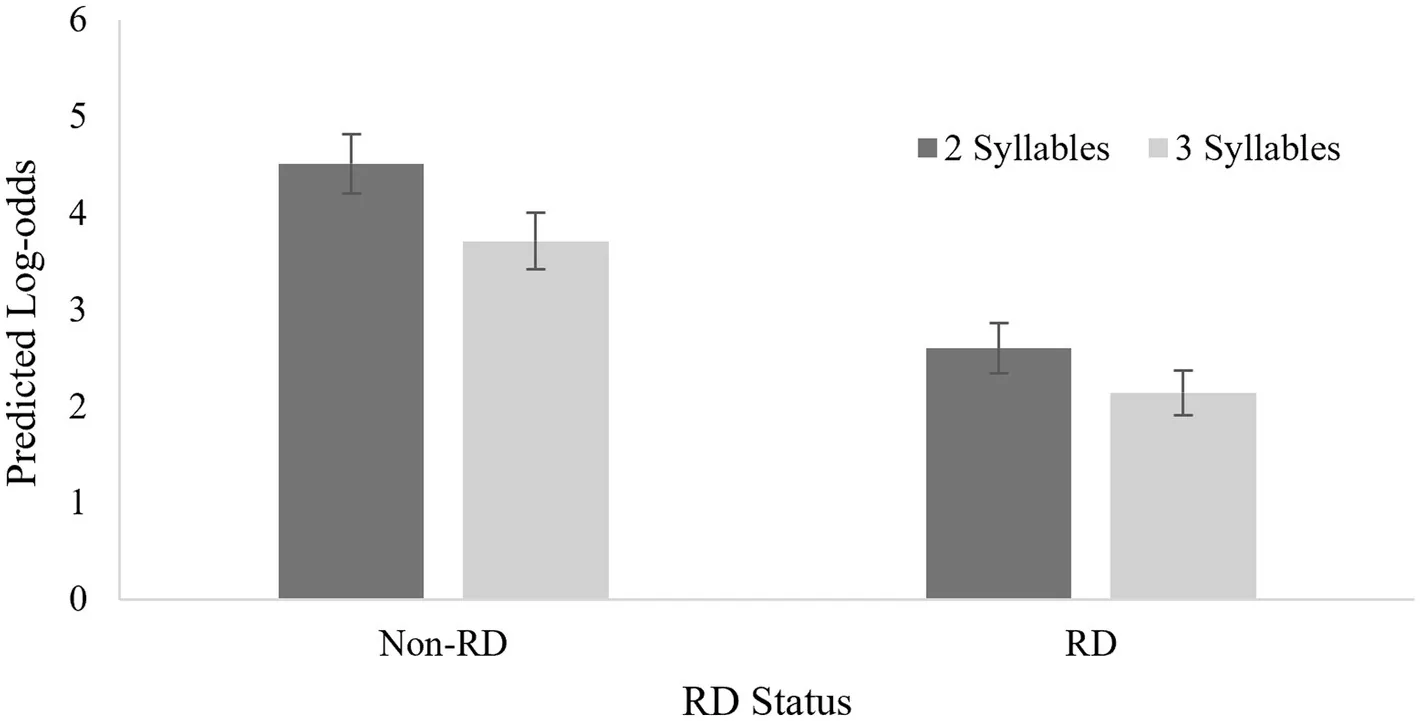
Interaction between number of syllables and RD status for fluency.

For the fourth question, we examined the interactions between the number of syllables and grade and the interactions between the number of syllables and RD status, reported in Table 7. There were no significant interactions for accuracy. For onset latency, there was a significant interaction between the number of syllables and both grade and RD status. The grade interaction showed that the difference in response latency between two-syllable and three-syllable words decreased across grades in log-odds terms (see Figure 1). The RD-latency interaction showed that students with RD showed a smaller difference in latency between two- and three-syllable words than students without RD. That is, while students with RD began reading three-syllable words more slowly than two-syllable words, this latency difference was smaller for those students than their peers without RD, *b* = 0.24, *p* = 0.03 (see Figure 2). There was also an interaction between RD status and fluency that was similar to the latency effect: Students with RD read three-syllable words more slowly than two-syllable words but the difference between three- and two-syllable word reading fluency was smaller than for students without RD, *b* = 0.34, *p* < 0.001 (see Figure 3).

**Table 7:** Predicted probabilities from interaction models.

Variable	Fluency^a^		Onset latency^b^	
	2 Syllables	3 Syllables	2 Syllables	3 Syllables
RD status				
No RD	0.86	0.73	0.88	0.83
RD	0.47	0.35	0.48	0.43
Grade status				
Grade 2	—	—	0.89	0.82
Grade 3	—	—	0.94	0.91
Grade 4	—	—	0.96	0.96

^a^*b* = 0.34, *p* < 0.001. ^b^For the interaction of the syllable and the RD status, *b* = 0.24, *p* = 0.03. For the interaction of the syllable and grade, *b* = 0.22, *p* = 0.006.

## Discussion

To summarize the results, readers appeared to show sensitivity to the presence of syllables, and this was true for readers’ accuracy, fluency, and onset latency. The effects were robust, even after considering the effect of the number of letters and of the presence of known morphological units. All effects were negative: The number of syllables was negatively associated with accuracy, fluency, and onset latency—regardless of the number of letters or the presence of morphemes. We also observed interactions in onset latency by grade (decreasing difference between two- and three-syllable words across grades), onset latency by RD status (smaller difference between two- and three-syllable words for students with RD than for their typical peers), and fluency by RD status (smaller difference between two- and three-syllable words for students with RD than for their typical peers).

### Strength and importance of syllable effects

The findings of this study are particularly noteworthy because we tested possible syllable effects conservatively, namely using regression analyses that accounted for letter and morpheme effects. The persistent statistical significance of the syllable effect even after controlling for these factors strongly suggests that syllabic units have features unrelated to letters and morphemes that affect reader performance.

Although prior research on adults was ambiguous regarding the direction of the syllable effect when accounting for letter and morpheme factors, these effects are unambiguous: Syllables make processing more difficult, particularly if words are completely unknown and the readers are children in elementary school. No similar test has been conducted—even in regression studies with children where such an analysis might have been possible (i.e., Goodwin et al., 2013; Kearns, 2015).

#### Alternative hypothesis: vowel letter effects

Despite the strength of this result, we considered two alternative hypotheses that might compete with the finding observed. The first was the possibility that the syllable effect was associated with the number of vowel letters rather than the number of syllables. Vowel letters are powerful markers that may anchor readers’ perception of syllabic units. As a result, we tested the possibility that the number of vowel letters (i.e., *A, E, I, O, U,* or *Y* when not in a consonant position) rather than the number of syllables was driving this effect. For example, perhaps processing for the pseudoword *sweatle* involved attention specifically to the number of vowel letters (3 of these in *sweatle*) rather than information encoded by syllables that have both orthographic and phonological representations (2 of these in *sweatle*). We conducted analyses including covariates for the number of vowel letters and number of syllables. For accuracy, fluency, and onset latency, there was no significant effect for the number of vowel letters.

#### Alternative hypothesis: orthographic familiarity effects

We also considered the possibility that the syllable effect was related to orthographic pattern familiarity rather than the number of syllables itself. In prior studies, researchers observed that bigram frequency and syllable frequency were both associated with shorter response times in reading real Spanish words (e.g., Conrad et al., 2007). Conrad et al. (2007) concluded that their results were in part due to the reader’s familiarity with the syllable and orthographic string although they attributed this in part to a lexical access mechanism, an explanation with less relevance for this study of pseudoword reading. Nonetheless, frequently occurring multi-letter orthographic units would improve performance by providing information that is partially redundant with letter information but may serve to reduce inconsistencies in pronunciation. This was similar to the hypothesis we considered for the facilitative role of syllabic units in improving reading performance (although that hypothesis was strongly rejected).

In these analyses, we used log-transformed summed bigram frequency using words contained in a subset of words in Grade 1-Grade 5 texts in the *Educator’s Word Frequency Guide* (Zeno et al., 1995) as a covariate to capture orthographic familiarity. We did not include a syllable frequency covariate because syllable boundaries are often ambiguous in English (e.g., phonologically, *habit* follows CV phonology [Clements and Keyser, 1983] but orthographically is typically syllabified as *hab-it*) and because syllable frequency cannot account for the possibility of patterns that occur across syllables, especially important because many words lack clear syllabic boundaries in orthography (Seidenberg, 2016) or phonology (Treiman and Danis, 1988).

We observed a significant effect of bigram frequency in the onset latency model, with larger values for bigram frequency associated with longer onset latencies, in addition to a significant syllable effect (see Table 8). However, the negative effect falsified the familiarity-consistency effect we hypothesized. One possible explanation for this negative bigram frequency-latency effect is the potential for competition among words with similar orthographic patterns: The more similar a given pseudoword is to a known real word, the more time it will take for the reader to settle on a pronunciation for the pseudoword before beginning to say it. We do not have the data to explore whether this effect is also characteristic of monosyllabic words, but prior data align with this possibility (e.g., Andrews, 1997). As a result of these supplemental findings, we concluded that the syllabic features of English words themselves contain information that affects the reader’s ability to pronounce words quickly and accurately; it is not simply a question of orthographic familiarity. The negative bigram-frequency effect has additional relevance, suggesting that orthographic confusability presents a processing challenge for readers.

**Table 8:** Results of the regression analysis predicting onset latency.

Fixed effects	*b*	*SE*	*z*
Intercept	1.890***	0.313	6.042
Number of Syllables	−0.243*	0.112	−2.173
Number of Letters	−0.061	0.073	−0.833
Number of Vowels	−0.082	0.073	−1.125
Bigram Frequency	−0.316*	0.152	−2.075
Grade	0.711**	0.221	3.216
RD	−1.988***	0.325	−6.117
Random Effects	Variance		
Child	2.765		
Word	0.130		
Model Fit	Deviance		
	8,625		

Bigram Frequency was log-transformed mean-centered prior to analysis. *p < 0.05. **p < 0.01. ***p < 0.001.

### A mechanistic account of the syllable effect

We characterized our definition of a syllabic unit functionally without specifying the characteristics of an orthographic syllable that would explain it. The strength of these results warrants additional exploration of the possibilities. Existing data probably explain much of it. Vowel letters have notoriously unreliable pronunciations in English, and single-letter vowels (like *a* and *o* in *bacon*) are especially unreliable and frequent in polysyllabic words. They can have tense, lax, or reduced pronunciations in addition to all the variability in monosyllabic words (e.g., the sound of *a* in *want*, *ball* and *bat*) and a few other polysyllabic patterns like *i* in *glorious*.

In addition, the reader confronts the challenge of stress assignment. English has a tendency toward trochaic stress, but this is not reliable. Rastle and Coltheart’s (2000) attempt to characterize stress assignment using rules showed that stress assignment does not easily conform to a set of rules—even an expansive set. In other words, the lexical features of polysyllabic words do not increase consistency; they may reduce it.

Word length necessitates parsing unfamiliar words into more manageable units. These data suggest that the vowel-letter-anchored syllable structure is used for the task, in alignment with data from methodologically diverse studies (e.g., Hansen and Rodgers, 1965; Spoehr and Smith, 1973; Taft, 1992). This requires processing time. Perry et al. (2010) observed such a cost: The CDP++ model required more cycles to achieve a settled state for bisyllabic words than monosyllabic ones. Processing time further increases when syllable boundaries are ambiguous (e.g., what units to derive from *zebra*—a decision that curiously varies internationally—or *chaos*; Chetail and Content, 2012, 2014).

A notable further factor could be phonological. Planning a polysyllabic utterance requires processing not associated with orthography (Levelt et al., 1999), so it is possible that this could require processing time after the reader has identified the phonological form to produce for a given syllable. The present study does not address the time course of the interaction between print, phonology, and articulation, but this possibility is important to consider in any study examining syllable-level processing in word recognition. It is also very unlikely that the observed influence of the orthographic syllable on reading fluency would be mediated entirely by phonological factors. Further, even if syllabic phonology completely mediated the latency and fluency effects, this would unlikely extend to the accuracy finding.

### Contributions of syllable and morphological units

In terms of morphology, no researchers (save Carlisle and Stone, 2005—with a very different approach) have tried to separate syllable and morphological effects in children. This study is the first to show that readers show sensitivity to the syllabic structure of words even when accounting for the morphological structure. Moreover, the performance of the participants in this study (children in Grades 2, 3, and 4) was not associated with the presence of morphological units in the words in the models that also included syllable and letter covariates. The lack of morphological effects was surprising considering the importance of morphology in word reading shown in prior studies (Carlisle, 2010; Goodwin and Ahn, 2013; Kuo and Anderson, 2006); this result may be attributed to the role of morphological knowledge in reading over grades. The frequency with which derivational morphological units appear in English words increases dramatically over the elementary grades (Kearns et al., 2016; Nagy and Anderson, 1984). Thus, readers may not yet have encountered enough words with derivational morphemes to rely on such knowledge when reading polysyllabic words.

### Interactions with the syllable effect

A significant interaction with grade was only observed in onset latency, with a greater difference in onset latency between two- and three-syllable words in the earlier grades than the later ones. The difference suggests that these earlier-grade readers are progressing in a different way from students with RD with the same level of reading skill (reading-age matched) in that they show developing automaticity with syllabic structure while those with RD are limited in their capacity to use syllabic information and experience difficulty at all syllable lengths. The results revealed significant interactions between the syllable effect and RD status. These interactions were only observed in fluency and onset latency, but not in accuracy. Specifically, readers with typical achievement showed a more pronounced negative impact of adding a syllable in onset latency and fluency than students with RD, for whom two- and three-syllable words were similarly difficult. This suggests that for students with RD, even two-syllable pseudowords present such a high level of processing difficulty that performance does not further decrease as the number of syllables increases, in contrast to students with typical achievement who could handle the processing demands for two-syllable pseudowords. This RD effect was inconsistent with our hypothesis as performance of students with RD showed floor effects for two syllables instead of showing a bigger gap between two and three syllables. However, this result is consistent with the findings of Hautala et al. (2013) and phase theory in that students with RD have not yet established a strong enough representation of the sound-spelling patterns in the language at the simpler letter level to make any syllabic processing possible (see also Brown and Deavers, 1999).

### Implications for reading instruction

In terms of implications for education, recommendations are quite premature. This is the first and only study to explore the role of syllable, letter, and morpheme word features in children’s pronunciation of novel words, so replication would be important. In addition, this is not an experimental study contrasting the effects of instruction on syllabic units versus only letters or morphemes. However, the study does indicate that future reading curricula might benefit from the inclusion of practice locating and pronouncing syllables in polysyllabic words. Moreover, the results favor the continued use of instruction on syllables that characterizes some popular programs for poor readers (e.g., Clark-Edmands, 2012), although these data provide no further information to guide the design of instruction. These data align with prior studies in which researchers showed that a strategy involving vowel-based syllable identification in written polysyllabic words and pronouncing each syllable improves reading outcomes (O’Connor et al., 2015; Toste et al., 2019).

### Limitations

Although these results support the idea that a perception of a syllable-related unit affects word recognition performance, they do not provide evidence concerning the type of syllabic unit that facilitates fast and accurate pronunciation of unfamiliar words (e.g., BOSS, BOB, VCG) or any specific claims about the validity of the mechanistic account described above. Further studies of early readers’ syllabic processing would contribute to understanding of the reading acquisition process in alphabetic languages. This line of research could lead to innovations in reading instruction. If data continue to support the importance of syllable effects regardless of letter and morpheme factors, additional exploration of syllable strategies would be warranted.

Another important limitation of these results is that the onset latencies and response times were coded dichotomously. This strategy was employed for two reasons. The first was technical: The large number of items made it challenging for staff to obtain precise estimates for every item. A 2-s cutpoint reduced the need to calculate the precise length if the utterance began more than 2-s after presentation or lasted more than 2 s. The second concerned the distribution of responses. In word recognition studies including participants with dysfluencies (like the present study of performance of elementary-age students including some with RD), latency times are often positively skewed. This challenge can be mitigated with a linear transformation or by dichotomizing the outcome, as done here. The dichotomized outcome variables are an immutable feature of the dataset, so the reader must judge whether this decision invalidates any potential scientific merit. We judge that this is not the case because (a) the results provide converging evidence (i.e., supporting the accuracy finding) that syllables are perceptual units affecting all parts of the recognition process, (b) the presence of interactions indicates variability related to reader characteristics that merit further analysis, and (c) there are neither floor nor ceiling effects in performance (see Table 3).

However, a related limitation is that—despite the apparent variability in performance for latencies—the interactions may be attenuated by local ceiling effects for grade (Grade 4 students with typical achievement have very short latencies and high fluency and students with RD have much longer latencies and lower fluency). However, local performance for students with RD was not near the floor (i.e., most latencies and fluencies were not greater than 2 s), suggesting that the lack of variability related to RD (i.e., generally long onset latencies and low fluency regardless of the number of syllables) reflects difficulty that would likely extend to words with more syllables. In other words, elementary-age readers with RD would read words with many syllables as slowly as those with few syllables because any syllabic complexity reduces performance. Moreover, the fact that this interaction obtained across both outcomes further supports the idea that this is a meaningful result.

There are additional design-based limitations. One concerns the sample composition by grade and RD status. All Grade 2 students had typical achievement; Grade 3 and 4 students might have RD or not; and a subset of students, between the 25th and 35th percentile, was not included. Because of this design, we considered the possibility that grade and RD might interact in ways that would further complicate the interpretation of the interactions. To militate against this possibility, we conducted supplemental analyses including grade-by-RD interaction terms in all interaction models. We did not find a significant interaction between grade and RD and did not further explore grade-by-RD effects. Other technical limitations of the study include the fact that the data are cross-sectional rather than longitudinal and prevent direct claims about developmental changes and that the school-based context precluded the construction of a balanced sample. However, properly contextualized, these facts do not preclude interpretation of the results.

Taken together, these limitations suggest that, at a minimum, the main effects are robust and that there is some (perhaps tentative) evidence to support the conclusion that effects related to fluency and onset latency vary depending on RD and that onset latencies vary by grade. We think the interaction effects contribute meaningfully for the reasons already stated but acknowledge that readers should be careful to avoid overinterpreting this result. Replication will be essential for future studies using this approach.

## Conclusion

We set out to determine whether readers rely on information about the syllables in words as part of the process of reading unknown words. We found evidence that syllables are perceptual units in word recognition, even when we account for the fact that letter information is also relevant for word recognition accuracy, onset latency, and fluency. The results provide sufficient data to warrant the continued examination of syllable units in word reading studies—which requires, in turn, that word reading studies focus more on polysyllabic words. We hope this paper will push researchers to examine syllable effects in descriptive and experimental word reading studies focused on perception and education.

## Data Availability

The raw data supporting the conclusions of this article will be made available by the authors, without undue reservation.
