# Supplementary material for: Syllabic units in children’s word recognition
Source: Front Psychol. 2026 Sep 10;17:1852797. doi: 10.3389/fpsyg.2026.1852797 (PMC13601308; doi:10.3389/fpsyg.2026.1852797)
Supplement: Supplementary file 1 [file Data_Sheet_1.PDF]

Data Analysis _Table 6, 7, and 8

```
library(readxl)
library(lme4)
library(Matrix)
library(sjPlot)

setwd("G:/.shortcut-targets-by-id/1d4zBDGFV3X-D1RLjHpiT-vtaH5iPU73I/MSWR2Syll
/MSWR2Syll_Paper/3 Databases")

syll <- read_excel("mswr2_syllread_item_2026_05_18.xlsx")
syll[1:5, ]

## # A tibble: 5 × 13
##   word       wid   cid acc_corr fluency_nat on_lat_fast num_syll num_let
##   <chr>    <dbl> <dbl>    <dbl>       <dbl>       <dbl>    <dbl>   <dbl>
## 1 namigion  2296 20002        0           1           0        3       8
## 2 sweatle   2247 20002        1           1           0        2       7
## 3 dipation  2223 20002        0           1           1        3       8
## 4 mileome   2255 20002        1           1           1        2       7
## 5 sirber    2314 20002        0           0           0        2       6
## # ℹ 5 more variables: start_prefix <dbl>, end_suffix <dbl>, rd <dbl>,
## #   grade <dbl>, bigram_type_fbp <dbl>

syll$num_syll_2_int <- syll$num_syll - 2
syll$num_let_7_int <- syll$num_let - 7
syll$grade <- syll$grade - 2
syll$z_num_syll <- scale(syll$num_syll, center = TRUE, scale = TRUE)
syll$z_num_let <- scale(syll$num_let, center = TRUE, scale = TRUE)

#TABLE 6_SYLLABLE EFFECTS AND CHANGES IN RANDOM VARIABILITY ACROSS MOD
ELS

#6.1 ACCURACY MODELS

# MODEL 0: PERSON, ITEM
syll_acc0 <- glmer(acc_corr ~ 1 + grade + rd + (1|wid) + (1|cid),
                   data = syll, family = binomial("logit"), control = glmerCo
ntrol(optimizer="bobyqa"))
summary(syll_acc0)

## Generalized linear mixed model fit by maximum likelihood (Laplace
##   Approximation) [glmerMod]
##  Family: binomial  ( logit )
## Formula: acc_corr ~ 1 + grade + rd + (1 | wid) + (1 | cid)
##    Data: syll
## Control: glmerControl(optimizer = "bobyqa")
##
##       AIC       BIC    logLik -2*log(L)  df.resid
```

```
##   13216.4   13253.3   -6603.2   13206.4     11818
##
## Scaled residuals:
##     Min      1Q  Median      3Q     Max
## -4.5800 -0.6671 -0.3185  0.7388  7.6182
##
## Random effects:
##  Groups Name        Variance Std.Dev.
##  cid    (Intercept) 0.6062   0.7786
##  wid    (Intercept) 0.8371   0.9149
## Number of obs: 11823, groups:  cid, 123; wid, 98
##
## Fixed effects:
##             Estimate Std. Error z value Pr(>|z|)
## (Intercept)  -0.8236     0.1728  -4.767 1.87e-06 ***
## grade         0.6395     0.1037   6.164 7.08e-10 ***
## rd           -1.2216     0.1542  -7.920 2.38e-15 ***
## ---
## Signif. codes:  0 '***' 0.001 '**' 0.01 '*' 0.05 '.' 0.1 ' ' 1
##
## Correlation of Fixed Effects:
##       (Intr) grade
## grade -0.637
## rd    -0.197 -0.231
```

```
# MODEL 1: SYLLABLE
syll_acc1 <- glmer(acc_corr ~ num_syll_2_int + grade + rd + (1|wid) + (1|cid)
,
                   data = syll, family = binomial("logit"), control = glmerCo
ntrol(optimizer="bobyqa"))
summary(syll_acc1)
```

```
## Generalized linear mixed model fit by maximum likelihood (Laplace
##   Approximation) [glmerMod]
##  Family: binomial  ( logit )
## Formula: acc_corr ~ num_syll_2_int + grade + rd + (1 | wid) + (1 | cid)
##    Data: syll
## Control: glmerControl(optimizer = "bobyqa")
##
##       AIC       BIC    logLik -2*log(L)  df.resid
##   13202.9   13247.2   -6595.4   13190.9     11817
##
## Scaled residuals:
##     Min      1Q  Median      3Q     Max
## -4.5836 -0.6673 -0.3189  0.7381  7.7002
##
## Random effects:
##  Groups Name        Variance Std.Dev.
##  cid    (Intercept) 0.6061   0.7785
##  wid    (Intercept) 0.7074   0.8411
```

```
## Number of obs: 11823, groups:  cid, 123; wid, 98
##
## Fixed effects:
##                 Estimate Std. Error z value Pr(>|z|)
## (Intercept)      -0.5435     0.1820  -2.986  0.00282 **
## num_syll_2_int  -0.7413     0.1812  -4.090 4.31e-05 ***
## grade            0.6395     0.1037   6.165 7.05e-10 ***
## rd              -1.2217     0.1542  -7.921 2.36e-15 ***
## ---
## Signif. codes:  0 '***' 0.001 '**' 0.01 '*' 0.05 '.' 0.1 ' ' 1
##
## Correlation of Fixed Effects:
##             (Intr) nm__2_ grade
## nm_syll_2_n -0.373
## grade       -0.603 -0.004
## rd          -0.189  0.006 -0.231
```

```
# MODEL 2: SYLLABLE AND LETTER
syll_acc2 <- glmer(acc_corr ~ num_syll_2_int + num_let_7_int + grade + rd + (
1 | wid) + (1 | cid),
                   data = syll, family = binomial("logit"), control = glmerCo
ntrol(optimizer = "bobyqa"))
summary(syll_acc2)
```

```
## Generalized linear mixed model fit by maximum likelihood (Laplace
##   Approximation) [glmerMod]
##  Family: binomial  ( logit )
## Formula: acc_corr ~ num_syll_2_int + num_let_7_int + grade + rd + (1 |
##     wid) + (1 | cid)
##    Data: syll
## Control: glmerControl(optimizer = "bobyqa")
##
##       AIC       BIC    logLik -2*log(L)  df.resid
##   13199.2   13250.8   -6592.6   13185.2     11816
##
## Scaled residuals:
##     Min      1Q  Median      3Q     Max
## -4.6518 -0.6669 -0.3178  0.7401  7.6036
##
## Random effects:
##  Groups Name        Variance Std.Dev.
##  cid    (Intercept) 0.6060   0.7785
##  wid    (Intercept) 0.6648   0.8153
## Number of obs: 11823, groups:  cid, 123; wid, 98
##
## Fixed effects:
##                 Estimate Std. Error z value Pr(>|z|)
## (Intercept)      -0.5704     0.1804  -3.162  0.00157 **
## num_syll_2_int  -0.5234     0.1975  -2.650  0.00804 **
## num_let_7_int   -0.2713     0.1118  -2.427  0.01523 *
```

```
## grade            0.6396     0.1037   6.166 7.01e-10 ***
## rd              -1.2218     0.1542  -7.922 2.34e-15 ***
## ---
## Signif. codes:  0 '***' 0.001 '**' 0.01 '*' 0.05 '.' 0.1 ' ' 1
##
## Correlation of Fixed Effects:
##             (Intr) nm__2_ nm__7_ grade
## nm_syll_2_n -0.354
## num_lt_7_nt  0.063 -0.453
## grade       -0.608 -0.003 -0.003
## rd          -0.191  0.004  0.003 -0.231
```

```
# MODEL 3: SYLLABLE, LETTER, MORPHEME
syll_acc3 <- glmer(acc_corr ~ num_syll_2_int + num_let_7_int + start_prefix +
 end_suffix + grade + rd + (1 | wid) + (1 | cid),
                   data = syll, family = binomial("logit"), control = glmerCo
ntrol(optimizer = "bobyqa"))
summary(syll_acc3)
```

```
## Generalized linear mixed model fit by maximum likelihood (Laplace
##   Approximation) [glmerMod]
##  Family: binomial  ( logit )
## Formula:
## acc_corr ~ num_syll_2_int + num_let_7_int + start_prefix + end_suffix +
##     grade + rd + (1 | wid) + (1 | cid)
##    Data: syll
## Control: glmerControl(optimizer = "bobyqa")
##
##      AIC       BIC    logLik -2*log(L)  df.resid
##   13199.9   13266.3   -6591.0   13181.9     11814
##
## Scaled residuals:
##     Min      1Q  Median      3Q     Max
## -4.5931 -0.6662 -0.3177  0.7399  7.5694
##
## Random effects:
##  Groups Name        Variance Std.Dev.
##  cid    (Intercept) 0.6060   0.7785
##  wid    (Intercept) 0.6404   0.8002
## Number of obs: 11823, groups:  cid, 123; wid, 98
##
## Fixed effects:
##                Estimate Std. Error z value Pr(>|z|)
## (Intercept)    -0.48146    0.19756  -2.437  0.01481 *
## num_syll_2_int -0.49288    0.19731  -2.498  0.01249 *
## num_let_7_int  -0.29437    0.11345  -2.595  0.00947 **
## start_prefix   -0.46205    0.25929  -1.782  0.07475 .
## end_suffix     -0.07487    0.17850  -0.419  0.67490
## grade           0.63959    0.10372   6.166 6.99e-10 ***
## rd             -1.22177    0.15422  -7.922 2.34e-15 ***
```

```
## ---
## Signif. codes:  0 '***' 0.001 '**' 0.01 '*' 0.05 '.' 0.1 ' ' 1
##
## Correlation of Fixed Effects:
##             (Intr) nm__2_ nm__7_ strt_p end_sf grade
## nm_syll_2_n -0.237
## num_lt_7_nt  0.103 -0.405
## start_prefx -0.177 -0.052  0.154
## end_suffix  -0.388 -0.175 -0.191  0.039
## grade       -0.555 -0.003 -0.003 -0.002 -0.001
## rd          -0.175  0.003  0.004  0.002  0.001 -0.231
```

```
#Model Comparison
anova(syll_acc0, syll_acc1, syll_acc2, syll_acc3)
```

```
## Data: syll
## Models:
## syll_acc0: acc_corr ~ 1 + grade + rd + (1 | wid) + (1 | cid)
## syll_acc1: acc_corr ~ num_syll_2_int + grade + rd + (1 | wid) + (1 | cid)
## syll_acc2: acc_corr ~ num_syll_2_int + num_let_7_int + grade + rd + (1 | w
id) + (1 | cid)
## syll_acc3: acc_corr ~ num_syll_2_int + num_let_7_int + start_prefix + end_
suffix + grade + rd + (1 | wid) + (1 | cid)
##           npar   AIC   BIC  logLik -2*log(L)   Chisq Df Pr(>Chisq)
## syll_acc0    5 13216 13253 -6603.2     13206
## syll_acc1    6 13203 13247 -6595.4     13191 15.4646  1  8.407e-05 ***
## syll_acc2    7 13199 13251 -6592.6     13185  5.7189  1    0.01678 *
## syll_acc3    9 13200 13266 -6591.0     13182  3.2363  2    0.19826
## ---
## Signif. codes:  0 '***' 0.001 '**' 0.01 '*' 0.05 '.' 0.1 ' ' 1
```

```
#6.2 FLUENCY MODELS

# MODEL 0: PERSON, ITEM
syll_flu0 <- glmer(fluency_nat ~ 1 + grade + rd + (1|wid) + (1|cid),
                   data = syll, family = binomial("logit"), control = glmerCo
ntrol(optimizer="bobyqa"))
summary(syll_flu0)
```

```
## Generalized linear mixed model fit by maximum likelihood (Laplace
##   Approximation) [glmerMod]
##  Family: binomial  ( logit )
## Formula: fluency_nat ~ 1 + grade + rd + (1 | wid) + (1 | cid)
##    Data: syll
## Control: glmerControl(optimizer = "bobyqa")
##
##       AIC       BIC    logLik -2*log(L)  df.resid
##   10559.1   10596.0   -5274.6   10549.1     11818
##
## Scaled residuals:
##     Min      1Q  Median      3Q     Max
```

```
## -8.7124 -0.3789  0.2703  0.5000  4.9898
##
## Random effects:
##  Groups Name        Variance Std.Dev.
##  cid    (Intercept) 1.4776   1.2156
##  wid    (Intercept) 0.5731   0.7571
## Number of obs: 11823, groups:  cid, 123; wid, 98
##
## Fixed effects:
##             Estimate Std. Error z value Pr(>|z|)
## (Intercept)   1.3632     0.2361   5.773 7.79e-09 ***
## grade         0.7053     0.1608   4.386 1.16e-05 ***
## rd           -1.7534     0.2376  -7.381 1.57e-13 ***
## ---
## Signif. codes:  0 '***' 0.001 '**' 0.01 '*' 0.05 '.' 0.1 ' ' 1
##
## Correlation of Fixed Effects:
##       (Intr) grade
## grade -0.699
## rd    -0.230 -0.248
```

```
# MODEL 1: SYLLABLE
syll_flu1 <- glmer(fluency_nat ~ num_syll_2_int + grade + rd + (1|wid) + (1|c
id),
                   data = syll, family = binomial("logit"), control = glmerCo
ntrol(optimizer="bobyqa"))
summary(syll_flu1)
```

```
## Generalized linear mixed model fit by maximum likelihood (Laplace
##   Approximation) [glmerMod]
##  Family: binomial  ( logit )
## Formula: fluency_nat ~ num_syll_2_int + grade + rd + (1 | wid) + (1 |
 cid)
##    Data: syll
## Control: glmerControl(optimizer = "bobyqa")
##
##       AIC       BIC    logLik -2*log(L)  df.resid
##   10516.5   10560.8   -5252.3   10504.5     11817
##
## Scaled residuals:
##     Min      1Q  Median      3Q     Max
## -8.6274 -0.3773  0.2713  0.4994  4.8885
##
## Random effects:
##  Groups Name        Variance Std.Dev.
##  cid    (Intercept) 1.4753   1.2146
##  wid    (Intercept) 0.3394   0.5826
## Number of obs: 11823, groups:  cid, 123; wid, 98
##
## Fixed effects:
```

```
##                  Estimate Std. Error z value Pr(>|z|)    
## (Intercept)        1.7349     0.2366   7.333 2.26e-13 ***
## num_syll_2_int    -0.9858     0.1317  -7.483 7.27e-14 ***
## grade              0.7051     0.1607   4.388 1.15e-05 ***
## rd                -1.7531     0.2374  -7.385 1.52e-13 ***
## ---
## Signif. codes:  0 '***' 0.001 '**' 0.01 '*' 0.05 '.' 0.1 ' ' 1
## 
## Correlation of Fixed Effects:
##             (Intr) nm__2_ grade 
## nm_syll_2_n -0.219              
## grade       -0.696 -0.005       
## rd          -0.231  0.010 -0.248
```

```
# MODEL 2: SYLLABLE AND LETTER
syll_flu2 <- glmer(fluency_nat ~ num_syll_2_int + num_let_7_int + grade + rd
+ (1 | wid) + (1 | cid),
                   data = syll, family = binomial("logit"), control = glmerCo
ntrol(optimizer = "bobyqa"))
summary(syll_flu2)
```

```
## Generalized linear mixed model fit by maximum likelihood (Laplace
##   Approximation) [glmerMod]
##  Family: binomial  ( logit )
## Formula: fluency_nat ~ num_syll_2_int + num_let_7_int + grade + rd + (1 |

##     wid) + (1 | cid)
##    Data: syll
## Control: glmerControl(optimizer = "bobyqa")
## 
##       AIC       BIC    logLik -2*log(L)  df.resid 
##   10486.7   10538.3   -5236.3   10472.7     11816 
## 
## Scaled residuals: 
##     Min      1Q  Median      3Q     Max 
## -8.8303 -0.3741  0.2698  0.5004  4.9055 
## 
## Random effects:
##  Groups Name        Variance Std.Dev.
##  cid    (Intercept) 1.4749   1.2144  
##  wid    (Intercept) 0.2276   0.4771  
## Number of obs: 11823, groups:  cid, 123; wid, 98
## 
## Fixed effects:
##                  Estimate Std. Error z value Pr(>|z|)    
## (Intercept)       1.69122    0.23271   7.267 3.67e-13 ***
## num_syll_2_int -0.63351    0.12491  -5.072 3.94e-07 ***
## num_let_7_int  -0.43894    0.07179  -6.114 9.73e-10 ***
## grade           0.70535    0.16069   4.390 1.14e-05 ***
## rd             -1.75355    0.23736  -7.388 1.49e-13 ***
```

```
## ---
## Signif. codes:  0 '***' 0.001 '**' 0.01 '*' 0.05 '.' 0.1 ' ' 1
##
## Correlation of Fixed Effects:
##             (Intr) nm__2_ nm__7_ grade
## nm_syll_2_n -0.181
## num_lt_7_nt  0.020 -0.446
## grade       -0.708 -0.003 -0.005
## rd          -0.235  0.007  0.009 -0.248
```

```
# MODEL 3: SYLLABLE, LETTER, MORPHEME
syll_flu3 <- glmer(fluency_nat ~ num_syll_2_int + num_let_7_int + start_prefi
x + end_suffix + grade + rd + (1 | wid) + (1 | cid),
                   data = syll, family = binomial("logit"), control = glmerCo
ntrol(optimizer = "bobyqa"))
summary(syll_flu3)
```

```
## Generalized linear mixed model fit by maximum likelihood (Laplace
##   Approximation) [glmerMod]
##  Family: binomial  ( logit )
## Formula: fluency_nat ~ num_syll_2_int + num_let_7_int + start_prefix +
##     end_suffix + grade + rd + (1 | wid) + (1 | cid)
##    Data: syll
## Control: glmerControl(optimizer = "bobyqa")
##
##       AIC       BIC    logLik -2*log(L)  df.resid
##   10489.6   10556.0   -5235.8   10471.6     11814
##
## Scaled residuals:
##     Min      1Q  Median      3Q     Max
## -8.9303 -0.3745  0.2694  0.5002  4.8916
##
## Random effects:
##  Groups Name        Variance Std.Dev.
##  cid    (Intercept) 1.4747   1.2144
##  wid    (Intercept) 0.2243   0.4736
## Number of obs: 11823, groups:  cid, 123; wid, 98
##
## Fixed effects:
##                Estimate Std. Error z value Pr(>|z|)
## (Intercept)     1.63804    0.23856   6.866 6.59e-12 ***
## num_syll_2_int -0.65768    0.12639  -5.204 1.95e-07 ***
## num_let_7_int  -0.45280    0.07373  -6.141 8.18e-10 ***
## start_prefix    0.02109    0.16783   0.126    0.900
## end_suffix      0.12041    0.11560   1.042    0.298
## grade           0.70529    0.16068   4.389 1.14e-05 ***
## rd             -1.75343    0.23735  -7.387 1.50e-13 ***
## ---
## Signif. codes:  0 '***' 0.001 '**' 0.01 '*' 0.05 '.' 0.1 ' ' 1
##
```

```
## Correlation of Fixed Effects:
##             (Intr) nm__2_ nm__7_ strt_p end_sf grade
## nm_syll_2_n -0.131
## num_lt_7_nt  0.045 -0.394
## start_prefx -0.094 -0.045  0.148
## end_suffix  -0.204 -0.182 -0.196  0.032
## grade       -0.691 -0.003 -0.005  0.000  0.000
## rd          -0.229  0.007  0.008 -0.001 -0.001 -0.248

# Model Comparison
anova(syll_flu0, syll_flu1, syll_flu2, syll_flu3)

## Data: syll
## Models:
## syll_flu0: fluency_nat ~ 1 + grade + rd + (1 | wid) + (1 | cid)
## syll_flu1: fluency_nat ~ num_syll_2_int + grade + rd + (1 | wid) + (1 | ci
d)
## syll_flu2: fluency_nat ~ num_syll_2_int + num_let_7_int + grade + rd + (1
| wid) + (1 | cid)
## syll_flu3: fluency_nat ~ num_syll_2_int + num_let_7_int + start_prefix + e
nd_suffix + grade + rd + (1 | wid) + (1 | cid)
##           npar   AIC   BIC  logLik -2*log(L)   Chisq Df Pr(>Chisq)
## syll_flu0    5 10559 10596 -5274.6     10549
## syll_flu1    6 10516 10561 -5252.3     10504 44.5760  1  2.447e-11 ***
## syll_flu2    7 10487 10538 -5236.3     10473 31.8570  1  1.660e-08 ***
## syll_flu3    9 10490 10556 -5235.8     10472  1.0832  2     0.5818
## ---
## Signif. codes:  0 '***' 0.001 '**' 0.01 '*' 0.05 '.' 0.1 ' ' 1

#6.3 ONSET LATENCY MODELS

# MODEL 0: PERSON, ITEM
syll_onlat0 <- glmer(on_lat_fast ~ 1 + grade + rd + (1|wid) + (1|cid),
                     data = syll, family = binomial("logit"), control = glmer
Control(optimizer="bobyqa"))
summary(syll_onlat0)

## Generalized linear mixed model fit by maximum likelihood (Laplace
##   Approximation) [glmerMod]
##  Family: binomial  ( logit )
## Formula: on_lat_fast ~ 1 + grade + rd + (1 | wid) + (1 | cid)
##    Data: syll
## Control: glmerControl(optimizer = "bobyqa")
##
##       AIC       BIC    logLik -2*log(L)  df.resid
##    8662.4    8699.0   -4326.2    8652.4     11091
##
## Scaled residuals:
##     Min      1Q  Median      3Q     Max
## -9.3817  0.0710  0.2101  0.4348  4.3293
##
```

```
## Random effects:
##  Groups Name        Variance Std.Dev.
##  cid    (Intercept) 2.7643   1.6626
##  wid    (Intercept) 0.1983   0.4453
## Number of obs: 11096, groups:  cid, 123; wid, 92
##
## Fixed effects:
##             Estimate Std. Error z value Pr(>|z|)
## (Intercept)   1.8139     0.3092   5.866 4.46e-09 ***
## grade         0.7114     0.2211   3.217   0.0013 **
## rd           -1.9879     0.3250  -6.117 9.56e-10 ***
## ---
## Signif. codes:  0 '***' 0.001 '**' 0.01 '*' 0.05 '.' 0.1 ' ' 1
##
## Correlation of Fixed Effects:
##       (Intr) grade
## grade -0.729
## rd    -0.239 -0.253
```

```
# MODEL 1: SYLLABLE
syll_onlat1 <- glmer(on_lat_fast ~ num_syll_2_int + grade + rd + (1|wid) + (1
|cid),
                     data = syll, family = binomial("logit"), control = glmer
Control(optimizer="bobyqa"))
summary(syll_onlat1)
```

```
## Generalized linear mixed model fit by maximum likelihood (Laplace
##   Approximation) [glmerMod]
##  Family: binomial  ( logit )
## Formula: on_lat_fast ~ num_syll_2_int + grade + rd + (1 | wid) + (1 |
 cid)
##    Data: syll
## Control: glmerControl(optimizer = "bobyqa")
##
##       AIC       BIC    logLik -2*log(L)  df.resid
##    8647.8    8691.7   -4317.9    8635.8     11090
##
## Scaled residuals:
##     Min      1Q  Median      3Q     Max
## -9.3986  0.0698  0.2085  0.4363  4.4393
##
## Random effects:
##  Groups Name        Variance Std.Dev.
##  cid    (Intercept) 2.7644   1.6626
##  wid    (Intercept) 0.1531   0.3913
## Number of obs: 11096, groups:  cid, 123; wid, 92
##
## Fixed effects:
##                Estimate Std. Error z value Pr(>|z|)
## (Intercept)      1.9783     0.3110   6.360 2.01e-10 ***
```

```
## num_syll_2_int  -0.4325     0.1013  -4.268 1.97e-05 ***
## grade            0.7114     0.2211   3.217   0.0013 **
## rd              -1.9882     0.3250  -6.117 9.51e-10 ***
## ---
## Signif. codes:  0 '***' 0.001 '**' 0.01 '*' 0.05 '.' 0.1 ' ' 1
##
## Correlation of Fixed Effects:
##             (Intr) nm__2_ grade
## nm_syll_2_n -0.129
## grade       -0.725 -0.002
## rd          -0.238  0.005 -0.253
```

```
# MODEL 2: SYLLABLE AND LETTER
syll_onlat2 <- glmer(on_lat_fast ~ num_syll_2_int + num_let_7_int + grade + r
d + (1 | wid) + (1 | cid),
                     data = syll, family = binomial("logit"), control = glmer
Control(optimizer = "bobyqa"))
summary(syll_onlat2)
```

```
## Generalized linear mixed model fit by maximum likelihood (Laplace
##   Approximation) [glmerMod]
##  Family: binomial  ( logit )
## Formula: on_lat_fast ~ num_syll_2_int + num_let_7_int + grade + rd + (1 |

##     wid) + (1 | cid)
##    Data: syll
## Control: glmerControl(optimizer = "bobyqa")
##
##      AIC      BIC   logLik -2*log(L)  df.resid
##   8644.1   8695.3  -4315.0    8630.1     11089
##
## Scaled residuals:
##     Min      1Q  Median      3Q     Max
## -9.5590  0.0701  0.2088  0.4371  4.4650
##
## Random effects:
##  Groups Name        Variance Std.Dev.
##  cid    (Intercept) 2.7654   1.6630
##  wid    (Intercept) 0.1408   0.3753
## Number of obs: 11096, groups:  cid, 123; wid, 92
##
## Fixed effects:
##                Estimate Std. Error z value Pr(>|z|)
## (Intercept)     1.95739    0.31083   6.297 3.03e-10 ***
## num_syll_2_int -0.30823    0.11084  -2.781  0.00542 **
## num_let_7_int  -0.15179    0.06258  -2.426  0.01529 *
## grade           0.71148    0.22118   3.217  0.00130 **
## rd             -1.98854    0.32507  -6.117 9.52e-10 ***
## ---
## Signif. codes:  0 '***' 0.001 '**' 0.01 '*' 0.05 '.' 0.1 ' ' 1
```

```
##
## Correlation of Fixed Effects:
##              (Intr) nm__2_ nm__7_ grade
## nm_syll_2_n -0.123
## num_lt_7_nt  0.024 -0.459
## grade       -0.725 -0.001 -0.001
## rd          -0.239  0.003  0.003 -0.253
```

```
# MODEL 3: SYLLABLE, LETTER, MORPHEME
syll_onlat3 <- glmer(on_lat_fast ~ num_syll_2_int + num_let_7_int + start_pre
fix + end_suffix + grade + rd + (1 | wid) + (1 | cid),
                    data = syll, family = binomial("logit"), control = glmer
Control(optimizer = "bobyqa"))
summary(syll_onlat3)
```

```
## Generalized linear mixed model fit by maximum likelihood (Laplace
##   Approximation) [glmerMod]
##  Family: binomial  ( logit )
## Formula: on_lat_fast ~ num_syll_2_int + num_let_7_int + start_prefix +
##     end_suffix + grade + rd + (1 | wid) + (1 | cid)
##    Data: syll
## Control: glmerControl(optimizer = "bobyqa")
##
##       AIC       BIC    logLik -2*log(L)  df.resid
##    8646.4    8712.2   -4314.2    8628.4     11087
##
## Scaled residuals:
##     Min      1Q  Median      3Q     Max
## -9.6387  0.0704  0.2084  0.4369  4.4656
##
## Random effects:
##  Groups Name        Variance Std.Dev.
##  cid    (Intercept) 2.7656   1.6630
##  wid    (Intercept) 0.1372   0.3704
## Number of obs: 11096, groups:  cid, 123; wid, 92
##
## Fixed effects:
##                Estimate Std. Error z value Pr(>|z|)
## (Intercept)     2.00909    0.31458   6.387 1.70e-10 ***
## num_syll_2_int -0.29225    0.11118  -2.629  0.00857 **
## num_let_7_int  -0.15395    0.06409  -2.402  0.01630 *
## start_prefix   -0.16202    0.14257  -1.136  0.25578
## end_suffix     -0.06959    0.10086  -0.690  0.49020
## grade           0.71150    0.22119   3.217  0.00130 **
## rd             -1.98854    0.32508  -6.117 9.53e-10 ***
## ---
## Signif. codes:  0 '***' 0.001 '**' 0.01 '*' 0.05 '.' 0.1 ' ' 1
##
## Correlation of Fixed Effects:
##              (Intr) nm__2_ nm__7_ strt_p end_sf grade
```

```
## nm_syll_2_n -0.096
## num_lt_7_nt  0.042 -0.415
## start_prefx -0.067 -0.043  0.148
## end_suffix  -0.143 -0.148 -0.197  0.042
## grade       -0.717 -0.001 -0.001  0.000 -0.001
## rd          -0.236  0.003  0.003  0.001  0.001 -0.253

# Model Comparison
anova(syll_onlat0, syll_onlat1, syll_onlat2, syll_onlat3)

## Data: syll
## Models:
## syll_onlat0: on_lat_fast ~ 1 + grade + rd + (1 | wid) + (1 | cid)
## syll_onlat1: on_lat_fast ~ num_syll_2_int + grade + rd + (1 | wid) + (1 |
cid)
## syll_onlat2: on_lat_fast ~ num_syll_2_int + num_let_7_int + grade + rd + (
1 | wid) + (1 | cid)
## syll_onlat3: on_lat_fast ~ num_syll_2_int + num_let_7_int + start_prefix +
 end_suffix + grade + rd + (1 | wid) + (1 | cid)
##             npar    AIC    BIC  logLik -2*log(L)   Chisq Df Pr(>Chisq)
## syll_onlat0    5 8662.4 8699.0 -4326.2    8652.4
## syll_onlat1    6 8647.8 8691.7 -4317.9    8635.8 16.5940  1   4.63e-05 ***
## syll_onlat2    7 8644.1 8695.3 -4315.0    8630.1  5.7160  1    0.01681 *
## syll_onlat3    9 8646.4 8712.2 -4314.2    8628.4  1.6815  2    0.43139
## ---
## Signif. codes:  0 '***' 0.001 '**' 0.01 '*' 0.05 '.' 0.1 ' ' 1

#ODDS RATIO TABLE
tab_model(syll_acc1, syll_acc2, syll_acc3,
          syll_flu1, syll_flu2, syll_flu3,
          syll_onlat1, syll_onlat2, syll_onlat3,
          dv.labels = c("Acc1", "Acc2", "Acc3",
                        "Flu1", "Flu2", "Flu3",
                        "Onlat1", "Onlat2", "Onlat3"),
          p.style = "stars",
          transform = "exp",
          show.re.var = TRUE,
          file = "model_table.doc")
```

	Acc1		Acc2		Acc3		Flu1		Flu2		Flu3		Onlat1		Onlat2		Onlat3	
Predictors	*Odds Ratios*	*CI*	*Odds Ratios*	*CI*	*Odds Ratios*	*CI*	*Odds Ratios*	*CI*	*Odds Ratios*	*CI*	*Odds Ratios*	*CI*	*Odds Ratios*	*CI*	*Odds Ratios*	*CI*	*Odds Ratios*	*CI*
(Intercept)	0.58 **	0.41 – 0.83	0.57 **	0.40 – 0.81	0.62 *	0.42 – 0.91	5.67 ***	3.57 – 9.01	5.43 ***	3.44 – 8.56	5.15 ***	3.22 – 8.21	7.23 ***	3.93 – 13.30	7.08 ***	3.85 – 13.02	7.46 ***	4.02 – 13.81
num syll 2 int	0.48 ***	0.33 – 0.68	0.59 **	0.40 – 0.87	0.61 *	0.41 – 0.90	0.37 ***	0.29 – 0.48	0.53 ***	0.42 – 0.68	0.52 ***	0.40 – 0.66	0.65 ***	0.53 – 0.79	0.73 **	0.59 – 0.91	0.75 **	0.60 – 0.93
grade	1.90 ***	1.55 – 2.32	1.90 ***	1.55 – 2.32	1.90 ***	1.55 – 2.32	2.02 ***	1.48 – 2.77	2.02 ***	1.48 – 2.77	2.02 ***	1.48 – 2.77	2.04 **	1.32 – 3.14	2.04 **	1.32 – 3.14	2.04 **	1.32 – 3.14
rd	0.29 ***	0.22 – 0.40	0.29 ***	0.22 – 0.40	0.29 ***	0.22 – 0.40	0.17 ***	0.11 – 0.28	0.17 ***	0.11 – 0.28	0.17 ***	0.11 – 0.28	0.14 ***	0.07 – 0.26	0.14 ***	0.07 – 0.26	0.14 ***	0.07 – 0.26
num let 7 int			0.76 *	0.61 – 0.95	0.74 **	0.60 – 0.93			0.64 ***	0.56 – 0.74	0.64 ***	0.55 – 0.73			0.86 *	0.76 – 0.97	0.86 *	0.76 – 0.97
start prefix					0.63	0.38 – 1.05					1.02	0.74 – 1.42					0.85	0.64 – 1.12
end suffix					0.93	0.65 – 1.32					1.13	0.90 – 1.41					0.93	0.77 – 1.14
**Random Effects**																		
σ^2	3.29		3.29		3.29		3.29		3.29		3.29		3.29		3.29		3.29	
τ_{00}	0.61 $_{cid}$		0.61 $_{cid}$		0.61 $_{cid}$		1.48 $_{cid}$		1.47 $_{cid}$		1.47 $_{cid}$		2.76 $_{cid}$		2.77 $_{cid}$		2.77 $_{cid}$	
	0.71 $_{wid}$		0.66 $_{wid}$		0.64 $_{wid}$		0.34 $_{wid}$		0.23 $_{wid}$		0.22 $_{wid}$		0.15 $_{wid}$		0.14 $_{wid}$		0.14 $_{wid}$	
ICC	0.29		0.28		0.27		0.36		0.34		0.34		0.47		0.47		0.47	
N	98 $_{wid}$		98 $_{wid}$		98 $_{wid}$		98 $_{wid}$		98 $_{wid}$		98 $_{wid}$		92 $_{wid}$		92 $_{wid}$		92 $_{wid}$	
	123 $_{cid}$		123 $_{cid}$		123 $_{cid}$		123 $_{cid}$		123 $_{cid}$		123 $_{cid}$		123 $_{cid}$		123 $_{cid}$		123 $_{cid}$	
Observations	11823		11823		11823		11823		11823		11823		11096		11096		11096	
Marginal R^2 / Conditional R^2	0.112 / 0.365		0.120 / 0.365		0.125 / 0.365		0.169 / 0.464		0.187 / 0.464		0.187 / 0.464		0.144 / 0.546		0.146 / 0.546		0.146 / 0.546	

* $p<0.05$ ** $p<0.01$ *** $p<0.001$

```
#TABLE 7_PREDICTED PROBABILITIES FROM INTERACTION MODELS

#7.1 ACCURACY

# MODEL 1: GRADE X SYLL INTERACTION
syll_acc_int1 <- glmer(acc_corr ~ num_syll_2_int + grade + rd + grade*num_syl
l_2_int + (1|wid) + (1|cid),
                       data = syll, family = binomial("logit"), control = glm
erControl(optimizer="bobyqa"))
summary(syll_acc_int1)

## Generalized linear mixed model fit by maximum likelihood (Laplace
##   Approximation) [glmerMod]
##  Family: binomial  ( logit )
## Formula: acc_corr ~ num_syll_2_int + grade + rd + grade * num_syll_2_int +

##     (1 | wid) + (1 | cid)
##    Data: syll
## Control: glmerControl(optimizer = "bobyqa")
##
##       AIC       BIC    logLik -2*log(L)  df.resid
##   13204.8   13256.5   -6595.4   13190.8     11816
##
## Scaled residuals:
##     Min      1Q  Median      3Q     Max
## -4.5987 -0.6671 -0.3187  0.7385  7.6853
##
## Random effects:
##  Groups Name        Variance Std.Dev.
##  cid    (Intercept) 0.6059   0.7784
##  wid    (Intercept) 0.7075   0.8412
## Number of obs: 11823, groups:  cid, 123; wid, 98
##
## Fixed effects:
##                      Estimate Std. Error z value Pr(>|z|)
## (Intercept)          -0.55105    0.18386  -2.997 0.002725 **
## num_syll_2_int       -0.71916    0.19680  -3.654 0.000258 ***
## grade                 0.64580    0.10600   6.093 1.11e-09 ***
## rd                   -1.22187    0.15421  -7.923 2.31e-15 ***
## num_syll_2_int:grade -0.01768    0.06135  -0.288 0.773243
## ---
## Signif. codes:  0 '***' 0.001 '**' 0.01 '*' 0.05 '.' 0.1 ' ' 1
##
## Correlation of Fixed Effects:
##             (Intr) nm__2_ grade  rd
## nm_syll_2_n -0.395
## grade       -0.613  0.077
## rd          -0.187  0.003 -0.227
## nm_syll_2_:  0.143 -0.390 -0.206  0.005
```

```r
# MODEL 2: RD X SYLL INTERACTION
syll_acc_int2 <- glmer(acc_corr ~ num_syll_2_int + grade + rd + rd*num_syll_2
_int + (1|wid) + (1|cid),
                        data = syll, family = binomial("logit"), control = glm
erControl(optimizer="bobyqa"))
summary(syll_acc_int2)
```

```
## Generalized linear mixed model fit by maximum likelihood (Laplace
##   Approximation) [glmerMod]
##  Family: binomial  ( logit )
## Formula: acc_corr ~ num_syll_2_int + grade + rd + rd * num_syll_2_int +
##     (1 | wid) + (1 | cid)
##    Data: syll
## Control: glmerControl(optimizer = "bobyqa")
##
##       AIC       BIC    logLik -2*log(L)  df.resid
##   13204.8   13256.4   -6595.4   13190.8     11816
##
## Scaled residuals:
##     Min      1Q  Median      3Q     Max
## -4.5980 -0.6668 -0.3193  0.7398  7.6388
##
## Random effects:
##  Groups Name        Variance Std.Dev.
##  cid    (Intercept) 0.6060   0.7785
##  wid    (Intercept) 0.7077   0.8412
## Number of obs: 11823, groups:  cid, 123; wid, 98
##
## Fixed effects:
##                     Estimate Std. Error z value Pr(>|z|)
## (Intercept)         -0.53932    0.18241  -2.957  0.00311 **
## num_syll_2_int      -0.75259    0.18433  -4.083 4.45e-05 ***
## grade                0.63960    0.10373   6.166 6.99e-10 ***
## rd                  -1.23257    0.15756  -7.823 5.17e-15 ***
## num_syll_2_int:rd    0.03183    0.09420   0.338  0.73547
## ---
## Signif. codes:  0 '***' 0.001 '**' 0.01 '*' 0.05 '.' 0.1 ' ' 1
##
## Correlation of Fixed Effects:
##             (Intr) nm__2_ grade  rd
## nm_syll_2_n -0.378
## grade       -0.601 -0.005
## rd          -0.199  0.042 -0.227
## nm_syll_2_:  0.068 -0.182  0.003 -0.205
```

```r
# MODEL 3: GRADE X SYLL INTERACTION (WITH LETTER)
syll_acc_int3 <- glmer(acc_corr ~ num_syll_2_int + num_let_7_int + grade + gr
ade*num_syll_2_int + (1 | wid) + (1 | cid),
                        data = syll, family = binomial("logit"), control = glm
```

```
erControl(optimizer = "bobyqa"))
summary(syll_acc_int3)
```

```
## Generalized linear mixed model fit by maximum likelihood (Laplace
##   Approximation) [glmerMod]
##  Family: binomial  ( logit )
## Formula:
## acc_corr ~ num_syll_2_int + num_let_7_int + grade + grade * num_syll_2_int
 +
##     (1 | wid) + (1 | cid)
##    Data: syll
## Control: glmerControl(optimizer = "bobyqa")
##
##      AIC       BIC    logLik -2*log(L)  df.resid
##   13250.3   13301.9   -6618.1   13236.3     11816
##
## Scaled residuals:
##     Min      1Q  Median      3Q     Max
## -4.6476 -0.6684 -0.3173  0.7426  7.5454
##
## Random effects:
##  Groups Name        Variance Std.Dev.
##  cid    (Intercept) 0.9582   0.9789
##  wid    (Intercept) 0.6652   0.8156
## Number of obs: 11823, groups:  cid, 123; wid, 98
##
## Fixed effects:
##                       Estimate Std. Error z value Pr(>|z|)
## (Intercept)           -0.86645    0.20657  -4.194 2.74e-05 ***
## num_syll_2_int        -0.50414    0.21210  -2.377 0.017459 *
## num_let_7_int         -0.27113    0.11181  -2.425 0.015312 *
## grade                  0.46142    0.12674   3.641 0.000272 ***
## num_syll_2_int:grade -0.01520    0.06152  -0.247 0.804919
## ---
## Signif. codes:  0 '***' 0.001 '**' 0.01 '*' 0.05 '.' 0.1 ' ' 1
##
## Correlation of Fixed Effects:
##             (Intr) nm__2_ nm__7_ grade
## nm_syll_2_n -0.334
## num_lt_7_nt  0.056 -0.423
## grade       -0.735  0.061 -0.002
## nm_syll_2_:  0.128 -0.364  0.003 -0.172
```

```
# MODEL 4: RD X SYLL INTERACTION (WITH LETTER)
syll_acc_int4 <- glmer(acc_corr ~ num_syll_2_int + num_let_7_int + grade + rd
 + rd*num_syll_2_int + (1 | wid) + (1 | cid),
                       data = syll, family = binomial("logit"), control = glm
erControl(optimizer = "bobyqa"))
summary(syll_acc_int4)
```

```
## Generalized linear mixed model fit by maximum likelihood (Laplace
##   Approximation) [glmerMod]
##  Family: binomial  ( logit )
## Formula: acc_corr ~ num_syll_2_int + num_let_7_int + grade + rd + rd *
##     num_syll_2_int + (1 | wid) + (1 | cid)
##    Data: syll
## Control: glmerControl(optimizer = "bobyqa")
##
##       AIC       BIC    logLik -2*log(L)  df.resid
##   13201.1   13260.1   -6592.5   13185.1     11815
##
## Scaled residuals:
##     Min      1Q  Median      3Q     Max
## -4.6666 -0.6665 -0.3185  0.7424  7.5420
##
## Random effects:
##  Groups Name        Variance Std.Dev.
##  cid    (Intercept) 0.6059   0.7784
##  wid    (Intercept) 0.6650   0.8155
## Number of obs: 11823, groups:  cid, 123; wid, 98
##
## Fixed effects:
##                     Estimate Std. Error z value Pr(>|z|)
## (Intercept)       -0.56620    0.18083  -3.131  0.00174 **
## num_syll_2_int    -0.53479    0.20026  -2.670  0.00758 **
## num_let_7_int     -0.27140    0.11180  -2.428  0.01520 *
## grade              0.63966    0.10372   6.167 6.96e-10 ***
## rd                -1.23285    0.15756  -7.825 5.08e-15 ***
## num_syll_2_int:rd  0.03239    0.09420   0.344  0.73092
## ---
## Signif. codes:  0 '***' 0.001 '**' 0.01 '*' 0.05 '.' 0.1 ' ' 1
##
## Correlation of Fixed Effects:
##             (Intr) nm__2_ nm__7_ grade  rd
## nm_syll_2_n -0.360
## num_lt_7_nt  0.063 -0.446
## grade       -0.607 -0.003 -0.003
## rd          -0.200  0.037  0.004 -0.227
## nm_syll_2_:  0.068 -0.166 -0.004  0.003 -0.205
```

```
# MODEL 5: GRADE X SYLL INTERACTION (WITH MORPH)
syll_acc_int5 <- glmer(acc_corr ~ num_syll_2_int + num_let_7_int + start_pref
ix + end_suffix + grade + grade*num_syll_2_int + (1 | wid) + (1 | cid),
                       data = syll, family = binomial("logit"), control = glm
erControl(optimizer = "bobyqa"))
summary(syll_acc_int5)
```

```
## Generalized linear mixed model fit by maximum likelihood (Laplace
##   Approximation) [glmerMod]
##  Family: binomial  ( logit )
```

```
## Formula:
## acc_corr ~ num_syll_2_int + num_let_7_int + start_prefix + end_suffix +
##     grade + grade * num_syll_2_int + (1 | wid) + (1 | cid)
##    Data: syll
## Control: glmerControl(optimizer = "bobyqa")
##
##      AIC       BIC    logLik -2*log(L)  df.resid
##   13251.0   13317.4   -6616.5   13233.0     11814
##
## Scaled residuals:
##     Min      1Q  Median      3Q     Max
## -4.5891 -0.6687 -0.3183  0.7418  7.5114
##
## Random effects:
##  Groups Name        Variance Std.Dev.
##  cid    (Intercept) 0.9582   0.9789
##  wid    (Intercept) 0.6408   0.8005
## Number of obs: 11823, groups:  cid, 123; wid, 98
##
## Fixed effects:
##                      Estimate Std. Error z value Pr(>|z|)
## (Intercept)          -0.77751    0.22169  -3.507 0.000453 ***
## num_syll_2_int       -0.47337    0.21196  -2.233 0.025525 *
## num_let_7_int        -0.29421    0.11348  -2.593 0.009524 **
## start_prefix         -0.46203    0.25935  -1.781 0.074834 .
## end_suffix           -0.07502    0.17855  -0.420 0.674355
## grade                 0.46153    0.12674   3.642 0.000271 ***
## num_syll_2_int:grade -0.01540    0.06152  -0.250 0.802402
## ---
## Signif. codes:  0 '***' 0.001 '**' 0.01 '*' 0.05 '.' 0.1 ' ' 1
##
## Correlation of Fixed Effects:
##             (Intr) nm__2_ nm__7_ strt_p end_sf grade
## nm_syll_2_n -0.240
## num_lt_7_nt  0.093 -0.378
## start_prefx -0.157 -0.049  0.154
## end_suffix  -0.346 -0.163 -0.191  0.039
## grade       -0.685  0.061 -0.002 -0.002  0.000
## nm_syll_2_:  0.119 -0.365  0.003  0.002  0.000 -0.172
```

```
# MODEL 6: RD X SYLL INTERACTION (WITH MORPH)
syll_acc_int6 <- glmer(acc_corr ~ num_syll_2_int + num_let_7_int + start_pref
ix + end_suffix + grade + rd + rd*num_syll_2_int + (1 | wid) + (1 | cid),
                       data = syll, family = binomial("logit"), control = glm
erControl(optimizer = "bobyqa"))
summary(syll_acc_int6)
```

```
## Generalized linear mixed model fit by maximum likelihood (Laplace
##   Approximation) [glmerMod]
##  Family: binomial  ( logit )
```

```
## Formula:
## acc_corr ~ num_syll_2_int + num_let_7_int + start_prefix + end_suffix +
##     grade + rd + rd * num_syll_2_int + (1 | wid) + (1 | cid)
##    Data: syll
## Control: glmerControl(optimizer = "bobyqa")
##
##      AIC       BIC    logLik -2*log(L)  df.resid
##   13201.8   13275.6   -6590.9   13181.8     11813
##
## Scaled residuals:
##     Min      1Q  Median      3Q     Max
## -4.6079 -0.6668 -0.3180  0.7407  7.5072
##
## Random effects:
##  Groups Name        Variance Std.Dev.
##  cid    (Intercept) 0.6059   0.7784
##  wid    (Intercept) 0.6406   0.8003
## Number of obs: 11823, groups:  cid, 123; wid, 98
##
## Fixed effects:
##                     Estimate Std. Error z value Pr(>|z|)
## (Intercept)         -0.47714    0.19795  -2.410  0.01594 *
## num_syll_2_int      -0.50446    0.20010  -2.521  0.01170 *
## num_let_7_int       -0.29454    0.11347  -2.596  0.00944 **
## start_prefix        -0.46238    0.25933  -1.783  0.07459 .
## end_suffix          -0.07485    0.17853  -0.419  0.67503
## grade                0.63969    0.10372   6.167 6.94e-10 ***
## rd                  -1.23302    0.15756  -7.826 5.04e-15 ***
## num_syll_2_int:rd    0.03287    0.09420   0.349  0.72712
## ---
## Signif. codes:  0 '***' 0.001 '**' 0.01 '*' 0.05 '.' 0.1 ' ' 1
##
## Correlation of Fixed Effects:
##             (Intr) nm__2_ nm__7_ strt_p end_sf grade  rd
## nm_syll_2_n -0.244
## num_lt_7_nt  0.102 -0.399
## start_prefx -0.177 -0.050  0.154
## end_suffix  -0.387 -0.173 -0.191  0.039
## grade       -0.554 -0.003 -0.003 -0.002 -0.001
## rd          -0.184  0.037  0.004  0.003  0.001 -0.227
## nm_syll_2_:  0.063 -0.166 -0.004 -0.004  0.000  0.003 -0.205

#7.2 FLUENCY

# MODEL 1: GRADE X SYLL INTERACTION
syll_flu_int1 <- glmer(fluency_nat ~ num_syll_2_int + grade + rd + grade*num_
syll_2_int + (1|wid) + (1|cid),
                      data = syll, family = binomial("logit"), control = glm
erControl(optimizer="bobyqa"))
summary(syll_flu_int1)
```

```
## Generalized linear mixed model fit by maximum likelihood (Laplace
##   Approximation) [glmerMod]
##  Family: binomial  ( logit )
## Formula: fluency_nat ~ num_syll_2_int + grade + rd + grade * num_syll_2_in
t +
##     (1 | wid) + (1 | cid)
##    Data: syll
## Control: glmerControl(optimizer = "bobyqa")
##
##       AIC       BIC    logLik -2*log(L)  df.resid
##   10518.5   10570.2   -5252.3   10504.5     11816
##
## Scaled residuals:
##     Min      1Q  Median      3Q     Max
## -8.6255 -0.3769  0.2710  0.4990  4.8864
##
## Random effects:
##  Groups Name        Variance Std.Dev.
##  cid    (Intercept) 1.4753   1.2146
##  wid    (Intercept) 0.3394   0.5826
## Number of obs: 11823, groups:  cid, 123; wid, 98
##
## Fixed effects:
##                          Estimate Std. Error z value Pr(>|z|)
## (Intercept)              1.731264   0.239193   7.238 4.56e-13 ***
## num_syll_2_int          -0.977817   0.152108  -6.428 1.29e-10 ***
## grade                    0.708530   0.164028   4.320 1.56e-05 ***
## rd                      -1.753376   0.237394  -7.386 1.51e-13 ***
## num_syll_2_int:grade -0.007096   0.067798  -0.105    0.917
## ---
## Signif. codes:  0 '***' 0.001 '**' 0.01 '*' 0.05 '.' 0.1 ' ' 1
##
## Correlation of Fixed Effects:
##             (Intr) nm__2_ grade  rd
## nm_syll_2_n -0.261
## grade       -0.704  0.096
## rd          -0.227  0.004 -0.244
## nm_syll_2_:  0.147 -0.500 -0.200  0.009
```

```
# MODEL 2: RD X SYLL INTERACTION
syll_flu_int2 <- glmer(fluency_nat ~ num_syll_2_int + grade + rd + rd*num_syl
l_2_int + (1|wid) + (1|cid),
                       data = syll, family = binomial("logit"), control = glm
erControl(optimizer="bobyqa"))
summary(syll_flu_int2)
```

```
## Generalized linear mixed model fit by maximum likelihood (Laplace
##   Approximation) [glmerMod]
##  Family: binomial  ( logit )
## Formula: fluency_nat ~ num_syll_2_int + grade + rd + rd * num_syll_2_int +
```

```
##     (1 | wid) + (1 | cid)
##    Data: syll
## Control: glmerControl(optimizer = "bobyqa")
##
##       AIC       BIC    logLik -2*log(L)  df.resid
##   10507.1   10558.8   -5246.6   10493.1     11816
##
## Scaled residuals:
##     Min      1Q  Median      3Q     Max
## -9.1177 -0.3882  0.2678  0.5027  4.5619
##
## Random effects:
##  Groups Name        Variance Std.Dev.
##  cid    (Intercept) 1.4746   1.2143
##  wid    (Intercept) 0.3416   0.5844
## Number of obs: 11823, groups:  cid, 123; wid, 98
##
## Fixed effects:
##                   Estimate Std. Error z value Pr(>|z|)
## (Intercept)         1.8232     0.2383   7.651 1.99e-14 ***
## num_syll_2_int     -1.1628     0.1421  -8.180 2.83e-16 ***
## grade               0.7096     0.1607   4.416 1.01e-05 ***
## rd                 -1.9168     0.2423  -7.910 2.58e-15 ***
## num_syll_2_int:rd   0.3459     0.1015   3.407 0.000658 ***
## ---
## Signif. codes:  0 '***' 0.001 '**' 0.01 '*' 0.05 '.' 0.1 ' ' 1
##
## Correlation of Fixed Effects:
##             (Intr) nm__2_ grade  rd
## nm_syll_2_n -0.245
## grade       -0.690 -0.009
## rd          -0.248  0.086 -0.245
## nm_syll_2_:  0.112 -0.369  0.010 -0.201
```

```
# MODEL 3: GRADE X SYLL INTERACTION (WITH LETTER)
syll_flu_int3 <- glmer(fluency_nat ~ num_syll_2_int + num_let_7_int + grade +
 grade*num_syll_2_int + (1 | wid) + (1 | cid),
                       data = syll, family = binomial("logit"), control = glm
erControl(optimizer = "bobyqa"))
summary(syll_flu_int3)
```

```
## Generalized linear mixed model fit by maximum likelihood (Laplace
##   Approximation) [glmerMod]
##  Family: binomial  ( logit )
## Formula: fluency_nat ~ num_syll_2_int + num_let_7_int + grade + grade *
##     num_syll_2_int + (1 | wid) + (1 | cid)
##    Data: syll
## Control: glmerControl(optimizer = "bobyqa")
##
```

```
##       AIC       BIC    logLik -2*log(L)  df.resid
##   10532.0   10583.6   -5259.0   10518.0     11816
##
## Scaled residuals:
##     Min      1Q  Median      3Q     Max
## -8.7918 -0.3748  0.2709  0.5000  4.8756
##
## Random effects:
##  Groups Name        Variance Std.Dev.
##  cid    (Intercept) 2.2306   1.4935
##  wid    (Intercept) 0.2278   0.4773
## Number of obs: 11823, groups:  cid, 123; wid, 98
##
## Fixed effects:
##                       Estimate Std. Error z value Pr(>|z|)
## (Intercept)           1.298835   0.274197   4.737 2.17e-06 ***
## num_syll_2_int       -0.629142   0.146342  -4.299 1.71e-05 ***
## num_let_7_int        -0.438923   0.071817  -6.112 9.86e-10 ***
## grade                 0.430283   0.192060   2.240   0.0251 *
## num_syll_2_int:grade -0.003816   0.067724  -0.056   0.9551
## ---
## Signif. codes:  0 '***' 0.001 '**' 0.01 '*' 0.05 '.' 0.1 ' ' 1
##
## Correlation of Fixed Effects:
##             (Intr) nm__2_ nm__7_ grade
## nm_syll_2_n -0.198
## num_lt_7_nt  0.019 -0.381
## grade       -0.829  0.087 -0.002
## nm_syll_2_:  0.130 -0.521  0.001 -0.169
```

```
# MODEL 4: RD X SYLL INTERACTION (WITH LETTER)
syll_flu_int4 <- glmer(fluency_nat ~ num_syll_2_int + num_let_7_int + grade +
 rd + rd*num_syll_2_int + (1 | wid) + (1 | cid),
                       data = syll, family = binomial("logit"), control = glm
erControl(optimizer = "bobyqa"))
summary(syll_flu_int4)
```

```
## Generalized linear mixed model fit by maximum likelihood (Laplace
##   Approximation) [glmerMod]
##  Family: binomial  ( logit )
## Formula: fluency_nat ~ num_syll_2_int + num_let_7_int + grade + rd + rd *

##     num_syll_2_int + (1 | wid) + (1 | cid)
##    Data: syll
## Control: glmerControl(optimizer = "bobyqa")
##
##       AIC       BIC    logLik -2*log(L)  df.resid
##   10477.4   10536.4   -5230.7   10461.4     11815
##
## Scaled residuals:
```

```
##     Min      1Q  Median      3Q     Max 
## -9.3281 -0.3859  0.2669  0.5034  4.5830 
## 
## Random effects:
##  Groups Name        Variance Std.Dev.
##  cid    (Intercept) 1.4742   1.214   
##  wid    (Intercept) 0.2294   0.479   
## Number of obs: 11823, groups:  cid, 123; wid, 98
## 
## Fixed effects:
##                     Estimate Std. Error z value Pr(>|z|)    
## (Intercept)          1.77887    0.23440   7.589 3.23e-14 ***
## num_syll_2_int      -0.80913    0.13576  -5.960 2.52e-09 ***
## num_let_7_int       -0.43943    0.07203  -6.101 1.06e-09 ***
## grade                0.70985    0.16069   4.418 9.98e-06 ***
## rd                  -1.91634    0.24232  -7.908 2.61e-15 ***
## num_syll_2_int:rd    0.34405    0.10156   3.388 0.000705 ***
## ---
## Signif. codes:  0 '***' 0.001 '**' 0.01 '*' 0.05 '.' 0.1 ' ' 1
## 
## Correlation of Fixed Effects:
##             (Intr) nm__2_ nm__7_ grade  rd    
## nm_syll_2_n -0.211                            
## num_lt_7_nt  0.019 -0.410                     
## grade       -0.702 -0.007 -0.005              
## rd          -0.252  0.086  0.009 -0.245       
## nm_syll_2_:  0.113 -0.385 -0.004  0.010 -0.201
```

```
# MODEL 5: GRADE X SYLL INTERACTION (WITH MORPH)
syll_flu_int5 <- glmer(fluency_nat ~ num_syll_2_int + num_let_7_int + start_p
refix + end_suffix + grade + grade*num_syll_2_int + (1 | wid) + (1 | cid),
                       data = syll, family = binomial("logit"), control = glm
erControl(optimizer = "bobyqa"))
summary(syll_flu_int5)
```

```
## Generalized linear mixed model fit by maximum likelihood (Laplace
##   Approximation) [glmerMod]
##  Family: binomial  ( logit )
## Formula: fluency_nat ~ num_syll_2_int + num_let_7_int + start_prefix +
##     end_suffix + grade + grade * num_syll_2_int + (1 | wid) +      (1 | ci
d)
##    Data: syll
## Control: glmerControl(optimizer = "bobyqa")
## 
##       AIC       BIC    logLik -2*log(L)  df.resid 
##   10534.9   10601.3   -5258.4   10516.9     11814 
## 
## Scaled residuals: 
##     Min      1Q  Median      3Q     Max 
## -8.8913 -0.3743  0.2704  0.5007  4.8617 
```

```
##
## Random effects:
##  Groups Name        Variance Std.Dev.
##  cid    (Intercept) 2.2303   1.4934
##  wid    (Intercept) 0.2245   0.4738
## Number of obs: 11823, groups:  cid, 123; wid, 98
##
## Fixed effects:
##                        Estimate Std. Error z value Pr(>|z|)
## (Intercept)            1.245583   0.279196   4.461 8.15e-06 ***
## num_syll_2_int        -0.653210   0.147587  -4.426 9.60e-06 ***
## num_let_7_int         -0.452807   0.073750  -6.140 8.27e-10 ***
## start_prefix           0.020974   0.167880   0.125   0.9006
## end_suffix             0.120558   0.115630   1.043   0.2971
## grade                  0.430281   0.192050   2.240   0.0251 *
## num_syll_2_int:grade -0.003927   0.067722  -0.058   0.9538
## ---
## Signif. codes:  0 '***' 0.001 '**' 0.01 '*' 0.05 '.' 0.1 ' ' 1
##
## Correlation of Fixed Effects:
##             (Intr) nm__2_ nm__7_ strt_p end_sf grade
## nm_syll_2_n -0.161
## num_lt_7_nt  0.040 -0.339
## start_prefx -0.081 -0.039  0.148
## end_suffix  -0.175 -0.155 -0.196  0.032
## grade       -0.814  0.086 -0.002  0.000  0.000
## nm_syll_2_:  0.128 -0.516  0.001  0.001 -0.002 -0.169
```

```
# MODEL 6: RD X SYLL INTERACTION (WITH MORPH)
syll_flu_int6 <- glmer(fluency_nat ~ num_syll_2_int + num_let_7_int + start_p
refix + end_suffix + grade + rd + rd*num_syll_2_int + (1 | wid) + (1 | cid),
                       data = syll, family = binomial("logit"), control = glm
erControl(optimizer = "bobyqa"))
summary(syll_flu_int6)
```

```
## Generalized linear mixed model fit by maximum likelihood (Laplace
##   Approximation) [glmerMod]
##  Family: binomial  ( logit )
## Formula: fluency_nat ~ num_syll_2_int + num_let_7_int + start_prefix +
##     end_suffix + grade + rd + rd * num_syll_2_int + (1 | wid) +      (1 |
cid)
##    Data: syll
## Control: glmerControl(optimizer = "bobyqa")
##
##       AIC       BIC    logLik -2*log(L)  df.resid
##   10480.2   10554.0   -5230.1   10460.2     11813
##
## Scaled residuals:
##     Min      1Q  Median      3Q     Max
## -9.4378 -0.3859  0.2675  0.5038  4.5692
```

```
##
## Random effects:
##  Groups Name        Variance Std.Dev.
##  cid    (Intercept) 1.4741   1.2141
##  wid    (Intercept) 0.2259   0.4753
## Number of obs: 11823, groups:  cid, 123; wid, 98
##
## Fixed effects:
##                     Estimate Std. Error z value Pr(>|z|)
## (Intercept)          1.72463    0.24020   7.180 6.97e-13 ***
## num_syll_2_int      -0.83427    0.13717  -6.082 1.19e-09 ***
## num_let_7_int       -0.45378    0.07396  -6.136 8.48e-10 ***
## start_prefix         0.02022    0.16836   0.120 0.904397
## end_suffix           0.12367    0.11598   1.066 0.286287
## grade                0.70978    0.16068   4.417 9.99e-06 ***
## rd                  -1.91659    0.24231  -7.910 2.58e-15 ***
## num_syll_2_int:rd    0.34481    0.10156   3.395 0.000686 ***
## ---
## Signif. codes:  0 '***' 0.001 '**' 0.01 '*' 0.05 '.' 0.1 ' ' 1
##
## Correlation of Fixed Effects:
##             (Intr) nm__2_ nm__7_ strt_p end_sf grade  rd
## nm_syll_2_n -0.164
## num_lt_7_nt  0.044 -0.362
## start_prefx -0.094 -0.041  0.148
## end_suffix  -0.203 -0.171 -0.196  0.032
## grade       -0.685 -0.007 -0.005  0.000  0.000
## rd          -0.246  0.086  0.009  0.000 -0.003 -0.245
## nm_syll_2_:  0.109 -0.382 -0.006 -0.001  0.009  0.010 -0.201
```

```
#7.3 ONSET LATENCY

# MODEL 1: GRADE X SYLL INTERACTION
syll_onl_gr1 <- glmer(on_lat_fast ~ num_syll_2_int + grade + rd + grade*num_s
yll_2_int + (1|wid) + (1|cid),
                      data = syll, family = binomial("logit"), control = glme
rControl(optimizer="bobyqa"))
summary(syll_onl_gr1)
```

```
## Generalized linear mixed model fit by maximum likelihood (Laplace
##   Approximation) [glmerMod]
##  Family: binomial  ( logit )
## Formula: on_lat_fast ~ num_syll_2_int + grade + rd + grade * num_syll_2_in
t +
##     (1 | wid) + (1 | cid)
##    Data: syll
## Control: glmerControl(optimizer = "bobyqa")
##
##      AIC      BIC   logLik -2*log(L)  df.resid
##   8642.3   8693.5  -4314.2    8628.3     11089
```

```
##
## Scaled residuals:
##     Min      1Q  Median      3Q     Max
## -9.6628  0.0724  0.2092  0.4343  4.2286
##
## Random effects:
##  Groups Name        Variance Std.Dev.
##  cid    (Intercept) 2.7667   1.6633
##  wid    (Intercept) 0.1535   0.3918
## Number of obs: 11096, groups:  cid, 123; wid, 92
##
## Fixed effects:
##                      Estimate Std. Error z value Pr(>|z|)
## (Intercept)           2.08251    0.31368   6.639 3.16e-11 ***
## num_syll_2_int       -0.67204    0.13370  -5.027 4.99e-07 ***
## grade                 0.61714    0.22385   2.757  0.00583 **
## rd                   -1.98661    0.32513  -6.110 9.95e-10 ***
## num_syll_2_int:grade  0.21785    0.07918   2.751  0.00594 **
## ---
## Signif. codes:  0 '***' 0.001 '**' 0.01 '*' 0.05 '.' 0.1 ' ' 1
##
## Correlation of Fixed Effects:
##             (Intr) nm__2_ grade  rd
## nm_syll_2_n -0.180
## grade       -0.730  0.101
## rd          -0.236  0.003 -0.250
## nm_syll_2_:  0.123 -0.651 -0.152  0.000
```

```
# MODEL 2: RD X SYLL INTERACTION
syll_onl_int2 <- glmer(on_lat_fast ~ num_syll_2_int + grade + rd + rd*num_syl
l_2_int + (1|wid) + (1|cid),
                      data = syll, family = binomial("logit"), control = glme
rControl(optimizer="bobyqa"))
summary(syll_onl_int2)
```

```
## Generalized linear mixed model fit by maximum likelihood (Laplace
##   Approximation) [glmerMod]
##  Family: binomial  ( logit )
## Formula: on_lat_fast ~ num_syll_2_int + grade + rd + rd * num_syll_2_int +

##     (1 | wid) + (1 | cid)
##    Data: syll
## Control: glmerControl(optimizer = "bobyqa")
##
##      AIC       BIC    logLik -2*log(L)  df.resid
##    8645.3    8696.5   -4315.7    8631.3     11089
##
## Scaled residuals:
##     Min      1Q  Median      3Q     Max
## -9.7076  0.0676  0.2084  0.4375  4.2672
```

```
##
## Random effects:
##  Groups Name        Variance Std.Dev.
##  cid    (Intercept) 2.7660   1.663
##  wid    (Intercept) 0.1537   0.392
## Number of obs: 11096, groups:  cid, 123; wid, 92
##
## Fixed effects:
##                     Estimate Std. Error z value Pr(>|z|)
## (Intercept)           2.0342     0.3124   6.512 7.40e-11 ***
## num_syll_2_int       -0.5588     0.1176  -4.751 2.03e-06 ***
## grade                 0.7131     0.2212   3.224  0.00127 **
## rd                   -2.0919     0.3288  -6.362 1.99e-10 ***
## num_syll_2_int:rd     0.2405     0.1130   2.128  0.03334 *
## ---
## Signif. codes:  0 '***' 0.001 '**' 0.01 '*' 0.05 '.' 0.1 ' ' 1
##
## Correlation of Fixed Effects:
##              (Intr) nm__2_ grade  rd
## nm_syll_2_n -0.156
## grade       -0.722 -0.004
## rd          -0.248  0.082 -0.251
## nm_syll_2_:  0.086 -0.506  0.004 -0.150
```

```
# MODEL 3: GRADE X SYLL INTERACTION (WITH LETTER)
syll_onl_int3 <- glmer(on_lat_fast ~ num_syll_2_int + num_let_7_int + grade +
 grade*num_syll_2_int + (1 | wid) + (1 | cid),
                       data = syll, family = binomial("logit"), control = glm
erControl(optimizer = "bobyqa"))
summary(syll_onl_int3)
```

```
## Generalized linear mixed model fit by maximum likelihood (Laplace
##   Approximation) [glmerMod]
##  Family: binomial  ( logit )
## Formula: on_lat_fast ~ num_syll_2_int + num_let_7_int + grade + grade *
##     num_syll_2_int + (1 | wid) + (1 | cid)
##    Data: syll
## Control: glmerControl(optimizer = "bobyqa")
##
##       AIC       BIC    logLik -2*log(L)  df.resid
##    8669.8    8721.0   -4327.9    8655.8     11089
##
## Scaled residuals:
##     Min      1Q  Median      3Q     Max
## -9.7971  0.0750  0.2095  0.4345  4.2322
##
## Random effects:
##  Groups Name        Variance Std.Dev.
##  cid    (Intercept) 3.6685   1.9153
##  wid    (Intercept) 0.1413   0.3759
```

```
## Number of obs: 11096, groups:  cid, 123; wid, 92
## 
## Fixed effects:
##                      Estimate Std. Error z value Pr(>|z|)    
## (Intercept)           1.62546    0.34633   4.693 2.69e-06 ***
## num_syll_2_int       -0.54859    0.14113  -3.887 0.000101 ***
## num_let_7_int        -0.15148    0.06266  -2.417 0.015631 *  
## grade                 0.29111    0.24650   1.181 0.237612    
## num_syll_2_int:grade  0.21796    0.07905   2.757 0.005826 ** 
## ---
## Signif. codes:  0 '***' 0.001 '**' 0.01 '*' 0.05 '.' 0.1 ' ' 1
## 
## Correlation of Fixed Effects:
##             (Intr) nm__2_ nm__7_ grade 
## nm_syll_2_n -0.157                     
## num_lt_7_nt  0.023 -0.362              
## grade       -0.843  0.088 -0.001       
## nm_syll_2_:  0.111 -0.618  0.001 -0.138

# MODEL 4: RD X SYLL INTERACTION (WITH LETTER)
syll_onl_int4 <- glmer(on_lat_fast ~ num_syll_2_int + num_let_7_int + grade + 
 rd + rd*num_syll_2_int + (1 | wid) + (1 | cid), 
                       data = syll, family = binomial("logit"), control = glm
erControl(optimizer = "bobyqa"))
summary(syll_onl_int4)

## Generalized linear mixed model fit by maximum likelihood (Laplace
##   Approximation) [glmerMod]
##  Family: binomial  ( logit )
## Formula: on_lat_fast ~ num_syll_2_int + num_let_7_int + grade + rd + rd *

##     num_syll_2_int + (1 | wid) + (1 | cid)
##    Data: syll
## Control: glmerControl(optimizer = "bobyqa")
## 
##      AIC      BIC   logLik -2*log(L)  df.resid 
##   8641.6   8700.1  -4312.8    8625.6     11088 
## 
## Scaled residuals: 
##     Min      1Q  Median      3Q     Max 
## -9.8738  0.0679  0.2084  0.4367  4.2915 
## 
## Random effects:
##  Groups Name        Variance Std.Dev.
##  cid    (Intercept) 2.7670   1.663   
##  wid    (Intercept) 0.1414   0.376   
## Number of obs: 11096, groups:  cid, 123; wid, 92
## 
## Fixed effects:
##                   Estimate Std. Error z value Pr(>|z|)
```

```
## (Intercept)        2.01329    0.31216   6.450 1.12e-10 ***
## num_syll_2_int    -0.43439    0.12586  -3.451 0.000558 ***
## num_let_7_int     -0.15211    0.06267  -2.427 0.015213 *
## grade              0.71318    0.22125   3.223 0.001267 **
## rd                -2.09237    0.32888  -6.362 1.99e-10 ***
## num_syll_2_int:rd  0.24074    0.11303   2.130 0.033183 *
## ---
## Signif. codes:  0 '***' 0.001 '**' 0.01 '*' 0.05 '.' 0.1 ' ' 1
##
## Correlation of Fixed Effects:
##             (Intr) nm__2_ nm__7_ grade  rd
## nm_syll_2_n -0.150
## num_lt_7_nt  0.024 -0.403
## grade       -0.722 -0.004 -0.001
## rd          -0.248  0.076  0.003 -0.251
## nm_syll_2_:  0.086 -0.472 -0.003  0.004 -0.150
```

```
# MODEL 5: GRADE X SYLL INTERACTION (WITH MORPH)
syll_onl_int5 <- glmer(on_lat_fast ~ num_syll_2_int + num_let_7_int + start_p
refix + end_suffix + grade + grade*num_syll_2_int + (1 | wid) + (1 | cid),
                       data = syll, family = binomial("logit"), control = glm
erControl(optimizer = "bobyqa"))
summary(syll_onl_int5)
```

```
## Generalized linear mixed model fit by maximum likelihood (Laplace
##   Approximation) [glmerMod]
##  Family: binomial  ( logit )
## Formula: on_lat_fast ~ num_syll_2_int + num_let_7_int + start_prefix +
##     end_suffix + grade + grade * num_syll_2_int + (1 | wid) +      (1 | ci
d)
##    Data: syll
## Control: glmerControl(optimizer = "bobyqa")
##
##       AIC       BIC    logLik -2*log(L)  df.resid
##    8672.2    8738.0   -4327.1    8654.2     11087
##
## Scaled residuals:
##     Min      1Q  Median      3Q     Max
## -9.8658  0.0753  0.2087  0.4342  4.2328
##
## Random effects:
##  Groups Name        Variance Std.Dev.
##  cid    (Intercept) 3.6687   1.915
##  wid    (Intercept) 0.1376   0.371
## Number of obs: 11096, groups:  cid, 123; wid, 92
##
## Fixed effects:
##                        Estimate Std. Error z value Pr(>|z|)
## (Intercept)             1.67720    0.34970   4.796 1.62e-06 ***
## num_syll_2_int         -0.53261    0.14139  -3.767 0.000165 ***
```

```
## num_let_7_int        -0.15368    0.06417  -2.395 0.016627 *
## start_prefix         -0.16245    0.14274  -1.138 0.255105
## end_suffix           -0.06959    0.10099  -0.689 0.490777
## grade                 0.29114    0.24651   1.181 0.237582
## num_syll_2_int:grade  0.21797    0.07905   2.757 0.005827 **
## ---
## Signif. codes:  0 '***' 0.001 '**' 0.01 '*' 0.05 '.' 0.1 ' ' 1
##
## Correlation of Fixed Effects:
##             (Intr) nm__2_ nm__7_ strt_p end_sf grade
## nm_syll_2_n -0.137
## num_lt_7_nt  0.038 -0.328
## start_prefx -0.060 -0.033  0.148
## end_suffix  -0.129 -0.117 -0.197  0.042
## grade       -0.835  0.087 -0.001  0.000  0.000
## nm_syll_2_:  0.110 -0.616  0.001 -0.001  0.000 -0.138
```

```
# MODEL 6: RD X SYLL INTERACTION (WITH MORPH)
syll_onl_int6 <- glmer(on_lat_fast ~ num_syll_2_int + num_let_7_int + start_p
refix + end_suffix + grade + rd + rd*num_syll_2_int + (1 | wid) + (1 | cid),
                       data = syll, family = binomial("logit"), control = glm
erControl(optimizer = "bobyqa"))
summary(syll_onl_int6)
```

```
## Generalized linear mixed model fit by maximum likelihood (Laplace
##   Approximation) [glmerMod]
##  Family: binomial  ( logit )
## Formula: on_lat_fast ~ num_syll_2_int + num_let_7_int + start_prefix +
##     end_suffix + grade + rd + rd * num_syll_2_int + (1 | wid) +      (1 |
cid)
##    Data: syll
## Control: glmerControl(optimizer = "bobyqa")
##
##       AIC       BIC    logLik -2*log(L)  df.resid
##    8643.9    8717.1   -4312.0    8623.9     11086
##
## Scaled residuals:
##     Min      1Q  Median      3Q     Max
## -9.9556  0.0682  0.2079  0.4367  4.2922
##
## Random effects:
##  Groups Name        Variance Std.Dev.
##  cid    (Intercept) 2.7672   1.6635
##  wid    (Intercept) 0.1377   0.3711
## Number of obs: 11096, groups:  cid, 123; wid, 92
##
## Fixed effects:
##                   Estimate Std. Error z value Pr(>|z|)
## (Intercept)        2.06461    0.31590   6.536 6.33e-11 ***
## num_syll_2_int    -0.41841    0.12619  -3.316 0.000914 ***
```

```
## num_let_7_int     -0.15436    0.06418  -2.405 0.016171 *
## start_prefix      -0.16228    0.14279  -1.136 0.255760
## end_suffix        -0.06883    0.10102  -0.681 0.495632
## grade              0.71321    0.22125   3.223 0.001266 **
## rd                -2.09224    0.32889  -6.362 2.00e-10 ***
## num_syll_2_int:rd  0.24046    0.11303   2.127 0.033392 *
## ---
## Signif. codes:  0 '***' 0.001 '**' 0.01 '*' 0.05 '.' 0.1 ' ' 1
##
## Correlation of Fixed Effects:
##             (Intr) nm__2_ nm__7_ strt_p end_sf grade  rd
## nm_syll_2_n -0.125
## num_lt_7_nt  0.042 -0.365
## start_prefx -0.067 -0.037  0.148
## end_suffix  -0.143 -0.132 -0.197  0.041
## grade       -0.714 -0.003 -0.001  0.000 -0.001
## rd          -0.245  0.075  0.003  0.001  0.001 -0.251
## nm_syll_2_:  0.084 -0.471 -0.003 -0.001  0.003  0.004 -0.150
```

```
#PROBABILITY CALCULATION (RESULT SECTION)
#ACCURACY
print("[ACCURACY PROBABILITIES]")
```

```
## [1] "[ACCURACY PROBABILITIES]"
```

```
plogis(-0.493)
```

```
## [1] 0.3791871
```

```
start_logit_acc <- qlogis(0.38)
beta_syllable_acc <- -0.493
plogis(start_logit_acc + beta_syllable_acc)
```

```
## [1] 0.2723865
```

```
#FLUENCY
print("[FLUENCY PROBABILITIES]")
```

```
## [1] "[FLUENCY PROBABILITIES]"
```

```
plogis(-0.658)
```

```
## [1] 0.341189
```

```
start_logit_flu <- qlogis(0.34)
beta_syllable_flu <- -0.658
plogis(start_logit_flu + beta_syllable_flu)
```

```
## [1] 0.2106031
```

```
#ONSET LATENCY
print("[ONSET LATENCY PROBABILITIES]")
```

```
## [1] "[ONSET LATENCY PROBABILITIES]"
```

```r
plogis(-0.292)
```

```
## [1] 0.4275143
```

```r
start_logit_onlat <- qlogis(0.43)
beta_syllable_onlat <- -0.292
plogis(start_logit_onlat + beta_syllable_onlat)
```

```
## [1] 0.3603487
```

```r
#TABLE 8_RESULTS OF THE REGRESSION ANALYSIS PREDICTING ONSET LATENCY

# CALL LIBRARIES
library(lme4)
library(dplyr)
library(stringr)
library(Matrix)

# SET WORKING DIRECTORY
setwd("G:/.shortcut-targets-by-id/1d4zBDGFV3X-D1RLjHpiT-vtaH5iPU73I/MSWR2Syll
/MSWR2Syll_Paper/Frontiers Syllable Paper")

# IMPORT DATA
syll <- read.csv("mswr2_item_supplementary.csv", stringsAsFactors = FALSE)
syll[1:5, ]

##       word  wid   cid acc_corr fluency_nat on_lat_fast num_syll num_let rd
## 1 namigion 2296 20002        0           1           0        3       8  0
## 2  sweatle 2247 20002        1           1           0        2       7  0
## 3 dipation 2223 20002        0           1           1        3       8  0
## 4  mileome 2255 20002        1           1           1        2       7  0
## 5   sirber 2314 20002        0           0           0        2       6  0
##   grade bg_r_sum
## 1     3     3177
## 2     3     3231
## 3     3     4653
## 4     3     2916
## 5     3     3023

## PRESET
syll$num_syll_2_int <- syll$num_syll - 2
syll$num_let_7_int <- syll$num_let - 7
syll$grade <- syll$grade - 2
syll$z_num_syll <- scale(syll$num_syll, center = TRUE, scale = TRUE)
syll$z_num_let <- scale(syll$num_let, center = TRUE, scale = TRUE)
syll$num_vow <- str_count(tolower(as.character(syll$word)), "[aeiou]")
syll$num_vow_3_int <- syll$num_vow - 3
syll$log_bg_r_sum <- log(syll$bg_r_sum + 1)
syll$log_bg_r_sum_c <- scale(syll$log_bg_r_sum, center = TRUE, scale = FALSE)

syll_0 <- glmer(acc_corr ~ 1 + grade + rd + (1|wid) + (1|cid),
                data = syll, family = binomial("logit"),
                control = glmerControl(optimizer="bobyqa"))
summary(syll_0)

## Generalized linear mixed model fit by maximum likelihood (Laplace
##   Approximation) [glmerMod]
##  Family: binomial  ( logit )
## Formula: acc_corr ~ 1 + grade + rd + (1 | wid) + (1 | cid)
##    Data: syll
```

```
## Control: glmerControl(optimizer = "bobyqa")
##
##       AIC       BIC    logLik -2*log(L)  df.resid
##   13216.4   13253.3   -6603.2   13206.4     11818
##
## Scaled residuals:
##     Min      1Q  Median      3Q     Max
## -4.5800 -0.6671 -0.3185  0.7388  7.6182
##
## Random effects:
##  Groups Name        Variance Std.Dev.
##  cid    (Intercept) 0.6062   0.7786
##  wid    (Intercept) 0.8371   0.9149
## Number of obs: 11823, groups:  cid, 123; wid, 98
##
## Fixed effects:
##             Estimate Std. Error z value Pr(>|z|)
## (Intercept)  -0.8236     0.1728  -4.767 1.87e-06 ***
## grade         0.6395     0.1037   6.164 7.08e-10 ***
## rd           -1.2216     0.1542  -7.920 2.38e-15 ***
## ---
## Signif. codes:  0 '***' 0.001 '**' 0.01 '*' 0.05 '.' 0.1 ' ' 1
##
## Correlation of Fixed Effects:
##       (Intr) grade
## grade -0.637
## rd    -0.197 -0.231
```

```
#ACCURACY
syll_A <- glmer(acc_corr ~ num_syll_2_int + num_let_7_int + num_vow_3_int + l
og_bg_r_sum_c + grade + rd + (1|wid) + (1|cid),
                 data = syll, family = binomial("logit"),
                 control = glmerControl(optimizer="bobyqa"))
summary(syll_A)
```

```
## Generalized linear mixed model fit by maximum likelihood (Laplace
##   Approximation) [glmerMod]
##  Family: binomial  ( logit )
## Formula:
## acc_corr ~ num_syll_2_int + num_let_7_int + num_vow_3_int + log_bg_r_sum_c
 +
##     grade + rd + (1 | wid) + (1 | cid)
##    Data: syll
## Control: glmerControl(optimizer = "bobyqa")
##
##       AIC       BIC    logLik -2*log(L)  df.resid
##   13201.4   13267.8   -6591.7   13183.4     11814
##
## Scaled residuals:
##     Min      1Q  Median      3Q     Max
```

```
## -4.6481 -0.6666 -0.3176  0.7418  7.6072
##
## Random effects:
##  Groups Name        Variance Std.Dev.
##  cid    (Intercept) 0.6060   0.7785
##  wid    (Intercept) 0.6518   0.8073
## Number of obs: 11823, groups:  cid, 123; wid, 98
##
## Fixed effects:
##                  Estimate Std. Error z value Pr(>|z|)
## (Intercept)       -0.6444     0.1936  -3.328 0.000874 ***
## num_syll_2_int   -0.4959     0.2012  -2.464 0.013732 *
## num_let_7_int    -0.2330     0.1329  -1.754 0.079502 .
## num_vow_3_int    -0.1658     0.1328  -1.248 0.211877
## log_bg_r_sum_c    0.1153     0.2767   0.417 0.676989
## grade             0.6396     0.1037   6.166 7.00e-10 ***
## rd               -1.2218     0.1542  -7.922 2.33e-15 ***
## ---
## Signif. codes:  0 '***' 0.001 '**' 0.01 '*' 0.05 '.' 0.1 ' ' 1
##
## Correlation of Fixed Effects:
##             (Intr) nm__2_ nm__7_ nm__3_ lg____ grade
## nm_syll_2_n -0.396
## num_lt_7_nt -0.133 -0.238
## num_vw_3_nt  0.351 -0.169 -0.380
## lg_bg_r_sm_  0.133 -0.168 -0.416  0.039
## grade       -0.567 -0.002 -0.002 -0.001  0.000
## rd          -0.177  0.003  0.002  0.002 -0.001 -0.231
```

```
#FLUENCY
syll_B <- glmer(fluency_nat ~ num_syll_2_int + num_let_7_int + num_vow_3_int
+ log_bg_r_sum_c + grade + rd + (1|wid) + (1|cid),
                 data = syll, family = binomial("logit"),
                 control = glmerControl(optimizer="bobyqa"))
summary(syll_B)
```

```
## Generalized linear mixed model fit by maximum likelihood (Laplace
##   Approximation) [glmerMod]
##  Family: binomial  ( logit )
## Formula: fluency_nat ~ num_syll_2_int + num_let_7_int + num_vow_3_int +
##     log_bg_r_sum_c + grade + rd + (1 | wid) + (1 | cid)
##    Data: syll
## Control: glmerControl(optimizer = "bobyqa")
##
##       AIC       BIC    logLik -2*log(L)  df.resid
##   10489.2   10555.6   -5235.6   10471.2     11814
##
## Scaled residuals:
##     Min      1Q  Median      3Q     Max
## -8.8250 -0.3744  0.2695  0.5007  4.9190
```

```
##
## Random effects:
##  Groups Name        Variance Std.Dev.
##  cid    (Intercept) 1.4748   1.2144
##  wid    (Intercept) 0.2225   0.4717
## Number of obs: 11823, groups:  cid, 123; wid, 98
##
## Fixed effects:
##                  Estimate Std. Error z value Pr(>|z|)
## (Intercept)       1.74692    0.23714   7.367 1.75e-13 ***
## num_syll_2_int   -0.66922    0.12756  -5.246 1.55e-07 ***
## num_let_7_int    -0.49247    0.08529  -5.774 7.73e-09 ***
## num_vow_3_int     0.09342    0.08495   1.100    0.271
## log_bg_r_sum_c    0.10369    0.17695   0.586    0.558
## grade             0.70538    0.16069   4.390 1.13e-05 ***
## rd               -1.75353    0.23736  -7.388 1.49e-13 ***
## ---
## Signif. codes:  0 '***' 0.001 '**' 0.01 '*' 0.05 '.' 0.1 ' ' 1
##
## Correlation of Fixed Effects:
##             (Intr) nm__2_ nm__7_ nm__3_ lg____ grade
## nm_syll_2_n -0.214
## num_lt_7_nt -0.079 -0.228
## num_vw_3_nt  0.186 -0.179 -0.378
## lg_bg_r_sm_  0.072 -0.170 -0.419  0.048
## grade       -0.695 -0.004 -0.005  0.001  0.001
## rd          -0.231  0.007  0.008 -0.002 -0.001 -0.248
```

```
#ONSET LATENCY
syll_C <- glmer(on_lat_fast ~ num_syll_2_int + num_let_7_int + num_vow_3_int
+ log_bg_r_sum_c + grade + rd + (1|wid) + (1|cid),
                 data = syll, family = binomial("logit"),
                 control = glmerControl(optimizer="bobyqa"))
summary(syll_C)
```

```
## Generalized linear mixed model fit by maximum likelihood (Laplace
##   Approximation) [glmerMod]
##  Family: binomial  ( logit )
## Formula: on_lat_fast ~ num_syll_2_int + num_let_7_int + num_vow_3_int +
##     log_bg_r_sum_c + grade + rd + (1 | wid) + (1 | cid)
##    Data: syll
## Control: glmerControl(optimizer = "bobyqa")
##
##      AIC       BIC    logLik -2*log(L)  df.resid
##   8643.0    8708.8   -4312.5    8625.0     11087
##
## Scaled residuals:
##     Min      1Q  Median      3Q     Max
## -9.5583  0.0704  0.2088  0.4359  4.5495
##
```

```
## Random effects:
##  Groups Name        Variance Std.Dev.
##  cid    (Intercept) 2.7653   1.6629
##  wid    (Intercept) 0.1296   0.3601
## Number of obs: 11096, groups:  cid, 123; wid, 92
##
## Fixed effects:
##                 Estimate Std. Error z value Pr(>|z|)
## (Intercept)      1.89025    0.31287   6.042 1.52e-09 ***
## num_syll_2_int -0.24298    0.11182  -2.173   0.0298 *
## num_let_7_int  -0.06104    0.07328  -0.833   0.4049
## num_vow_3_int  -0.08230    0.07318  -1.125   0.2607
## log_bg_r_sum_c -0.31611    0.15231  -2.075   0.0379 *
## grade           0.71139    0.22118   3.216   0.0013 **
## rd             -1.98842    0.32506  -6.117 9.53e-10 ***
## ---
## Signif. codes:  0 '***' 0.001 '**' 0.01 '*' 0.05 '.' 0.1 ' ' 1
##
## Correlation of Fixed Effects:
##             (Intr) nm__2_ nm__7_ nm__3_ lg____ grade
## nm_syll_2_n -0.144
## num_lt_7_nt -0.041 -0.221
## num_vw_3_nt  0.117 -0.192 -0.391
## lg_bg_r_sm_  0.047 -0.196 -0.424  0.075
## grade       -0.721 -0.001 -0.001  0.000 -0.001
## rd          -0.237  0.003  0.001  0.001  0.002 -0.253
```
